# LINC00839 transcriptionally activated by ELK1 represses ferroptosis in nasopharyngeal carcinoma by regulating UPF1/RCHY1/DJ-1 axis

**DOI:** 10.1038/s41698-026-01467-1

**Published:** 2026-05-14

**Authors:** Feng Liu, Yu Li, Huai Liu, Ling Tang, ShuWen Fang, Xi Wang, Pan Chen, Hui Wang, MeiHua Bao, BinSheng He, Zhen Guo

**Affiliations:** 1 Hunan Key Laboratory of Translational Radiation Oncology, Department of Radiation Oncology, Hunan Cancer Hospital and The Affiliated Cancer Hospital of Xiangya School of Medicine, Central South University, Changsha, China; 2 Hunan Provincial Key Laboratory of the Fundamental and Clinical Research on Functional Nucleic Acid, the First Clinical College and the First Affiliated Hospital of Changsha Medical University, Changsha Medical University, Changsha, China; 3 Hunan Provincial Key Laboratory of the Research and Development of Novel Pharmaceutical Preparations, Changsha Medical University, Changsha, China; 4 Hunan Provincial Key Laboratory of the Fundamental and Clinical Research on Multimodal Oncology, the First Clinical College and the First Affiliated Hospital of Changsha Medical University, Changsha Medical University, Changsha, China

**Keywords:** Biochemistry, Cancer, Cell biology, Molecular biology, Oncology

## Abstract

Ferroptosis induction is a novel strategy for treating human cancers; however, the detailed mechanisms underlying ferroptosis resistance during nasopharyngeal carcinoma (NPC) progression remain unclear. Herein, we explored the role and potential mechanism of LINC00839 in ferroptosis resistance of NPC cells. We found that the expression levels of LINC00839 and transcription factor ets-like kinase 1 (ELK1) were elevated in NPC tissues, which were associated with a poor survival of NPC patients. Overexpression of LINC00839 or ELK1 reduced the sensitivity of NPC cells to ferroptosis-inducing drugs. Mechanistically, ELK1 directly bound to LINC00839 promoter to contribute to its transcription. Subsequently, LINC00839 destabilized ring finger and CHY zinc finger domain-containing 1 (RCHY1) mRNA through recruitment of up-frameshift 1 (UPF1), and consequently inhibited ubiquitination and degradation of DJ-1 protein. LINC00839 knockdown induced NPC cell ferroptosis, which was neutralized by RCHY1 depletion or DJ-1 overexpression. Knockdown of ELK1 or LINC00839 exerted synergistic roles with Erastin or ferroptosis-inducing chemotherapeutic drug Sorafenib to enhance ferroptosis, thereby delaying tumor growth in vivo. In summary, this study reveals that ELK1-mediated transcription activation of LINC00839 promotes ferroptosis resistance of NPC cells by destabilizing RCHY1 mRNA and subsequent repressing DJ-1 ubiquitination and degradation. These findings provide potential therapeutic targets for overcoming ferroptosis resistance in NPC.

## Introduction

Nasopharyngeal carcinoma (NPC) is an aggressive tumor that commonly occurs in Southern China and Southeast Asia^1^. Although the prognosis of NPC patients at an early stage is favorable due to the development of comprehensive therapies, most NPC patients at an advanced stage still suffer a high frequency of recurrence and distant metastasis^2,3^. Currently, relapse and metastasis remain the leading reasons for treatment failure and death of NPC patients^4^. Hence, uncovering the detailed mechanisms underlying NPC progression will be of great significance in developing novel therapeutic strategies.

Ferroptosis is a new type of cell death that is characterized by accumulated iron and lipid peroxidation^5^, which participates in the progression of multiple disorders, including NPC^6,7^. A previous study suggested that induction of ferroptosis caused NPC cell death, highlighting the therapeutic potential of ferroptosis-inducing drugs for delaying NPC progression^8^. DJ-1, also known as Parkinson’s protein 7 (PARK7), exerts a pivotal role in anti-oxidative stress^9^ and serves as an oncogene for various human cancers^10^. Cao et al. documented that DJ-1 functioned as a ferroptosis inhibitor, and inhibition of DJ-1 effectively sensitized tumor cells to ferroptosis-based anti-cancer therapy^11^. Notably, abnormal high expression of DJ-1 favored invasion and metastasis of NPC cells^12^. However, the influence of DJ-1 on ferroptosis resistance and its potential mechanisms during NPC development still need to be comprehensively studied.

Long non-coding RNAs (lncRNAs) without protein-coding ability have been proven to play key roles in multiple pathophysiological conditions^13–17^. Recently, several functional lncRNAs have been discovered to modulate the development and treatment of NPC by various mechanisms, such as regulating DNA damage repair, mitochondria function, chromatin remodeling, and sponging with miRNAs^18–22^. Moreover, the differentially expressed lncRNAs related with ferroptosis have been demonstrated to influence the prognosis of NPC patients^23^. LINC00839 has been demonstrated to accelerate the progression of multiple tumors^24–26^. Notably, LINC00839 conferred the proliferative and metastatic capacities of NPC cells via transcriptional activation of AOC1 by recruitment of TAF15^27^. However, whether LINC00839 can affect ferroptosis resistance of NPC cells has not been explored.

Transcription factor ets-like kinase 1 (ELK1) belongs to the ETS family, which controls the transcription and expression of its target genes^28^. For example, ELK1 could directly bind to the promoter of KIAA0101 to trigger its transcription, which promoted NPC cell growth and apoptosis inhibition^29^. Another study documented that ELK1 transcriptionally activated GPX4 to repress ferroptosis, thereby promoting the malignant development of endometrial carcinoma^30^. So far, the regulation and underlying mechanisms of ELK1 in ferroptosis of NPC cells have not been reported.

This study discovered that both ELK1 and LINC00839 were up-regulated in NPC tissues and cells, which reduced the sensitivity of NPC cells to ferroptosis inducers (Erastin and RSL3), whereas knockdown of ELK1/LINC00839 exerted synergistic effects with chemotherapeutic drug Sorafenib in delaying tumor growth in vivo via inducing ferroptosis. Mechanistically, ELK1 directly bound to LINC00839 promoter to promote its transcription. Moreover, LINC00839 recruited up-frameshift 1 (UPF1) to reduce ring finger and CHY zinc finger domain-containing 1 (RCHY1) mRNA stability, and subsequently inhibited ubiquitination and degradation of DJ-1. Thus, our results elucidate the novel oncogenic role of LINC00839 in NPC development via inducing ferroptosis resistance, which suggests LINC00839 as a novel therapeutic target for NPC.

## Results

### ELK1 and LINC00839 are highly expressed in NPC, indicating a lower survival rate

**Fig. 1:**
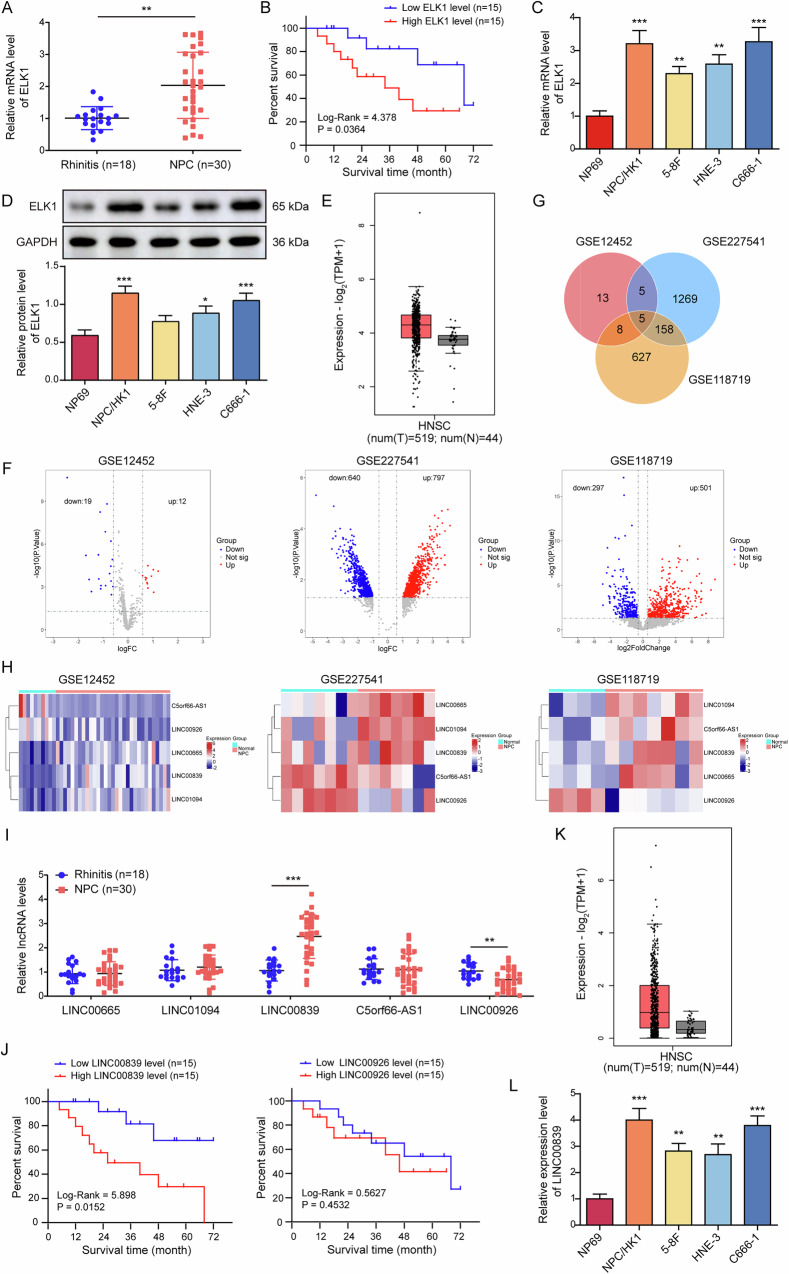
High expression of ELK1 and LINC00839 suggests a lower survival rate of NPC patients. **A** qRT-PCR analysis of ELK1 level in rhinitis tissues (*n* = 18) and NPC tissues (*n* = 30). **B** Survival rate of NPC patients with low (*n* = 15) or high (*n* = 15) expression of ELK1. **C**, **D** qRT-PCR and western blotting were used to detect the expression of ELK1 in multiple NPC cell lines and normal nasopharyngeal epithelial cells NP69. **E** GEPIA database evaluated the differential expression of ELK1 in head and neck squamous cell carcinoma (HNSC) (Tumor = 519, Normal = 44). **F** The differentially expressed lncRNAs in NPC tissues were screened out from datasets GSE12452, GSE227541, and GSE118719. The significant down-regulated and up-regulated lncRNAs were shown by volcano plots. **G** Five lncRNAs were screened out by overlapping the differentially expressed lncRNAs from the 3 datasets and shown in venn diagram. **H** The heat maps of these 5 differentially expressed lncRNAs in GSE12452, GSE227541, and GSE118719. **I** qRT-PCR analysis of the 5 differentially expressed lncRNAs in rhinitis tissues (*n* = 18) and NPC tissues (*n* = 30). **J** Survival rate of NPC patients with low (*n* = 15) or high (*n* = 15) expression of LINC00839 or LINC00926. **K** Differential expression of LINC00839 in HNSC was analyzed by GEPIA database (Tumor = 519, Normal = 44). **L** The expression of LINC00839 in NPC and NP69 cells was evaluated by qRT-PCR. *P < 0.05, **P < 0.01 and ***P < 0.001.

First, we collected 30 human NPC tissues and 18 rhinitis tissues to validate the abnormal expression of ELK1. As detected by qRT-PCR, ELK1 level was higher in NPC tissues as compared with rhinitis tissues (Fig. 1A). Notably, higher expression of ELK1 was correlated with a lower survival rate of NPC patients (Fig. 1B). Furthermore, ELK1 expression was measured in multiple NPC cell lines. Consistently, both mRNA (Fig. 1C) and protein (Fig. 1D) levels of ELK1 were higher in NPC cells than that in nasopharyngeal epithelial cell line NP69. GEPIA database also indicated that ELK1 was up-regulated in head and neck squamous cell carcinoma (HNSC) that contains NPC (Fig. 1E). Next, a series of datasets GSE12452, GSE227541, and GSE118719 from GEO database were used to evaluate the differentially expressed lncRNAs in NPC. The down-regulated and up-regulated lncRNAs were shown by volcano plots (Fig. 1F). By overlapping the data of 3 datasets, 5 differentially expressed lncRNAs including, LINC00665, LINC01094, LINC00839, C5orf66-AS1, and LINC00926 were screened out (Fig. 1G). The heat maps of these 5 differentially expressed lncRNAs in GSE12452, GSE227541, and GSE118719 were shown in Fig. 1H. Subsequently, we detected the expression of these 5 differentially expressed lncRNAs in the clinical NPC samples. We found that LINC00839 exhibited the highest level, while LINC00926 exhibited the lowest level in NPC tissues (Fig. 1I). Moreover, high expression of LINC00839 was correlated with a poorer survival, whereas LINC00926 expression was not associated with the survival of NPC patients (Fig. 1J). Therefore, LINC00839 was focused on in the following experiments. Furthermore, GEPIA database showed that LINC00839 was highly expressed in HNSC tissues (Fig. 1K). Consistently, up-regulation of LINC00839 was validated in multiple NPC cell lines (Fig. 1L). These findings suggested that high expression of ELK1 and LINC00839 in NPC indicated a poor prognosis of NPC patients.

### ELK1 directly binds to LINC00839 promoter to activate its transcription

**Fig. 2:**
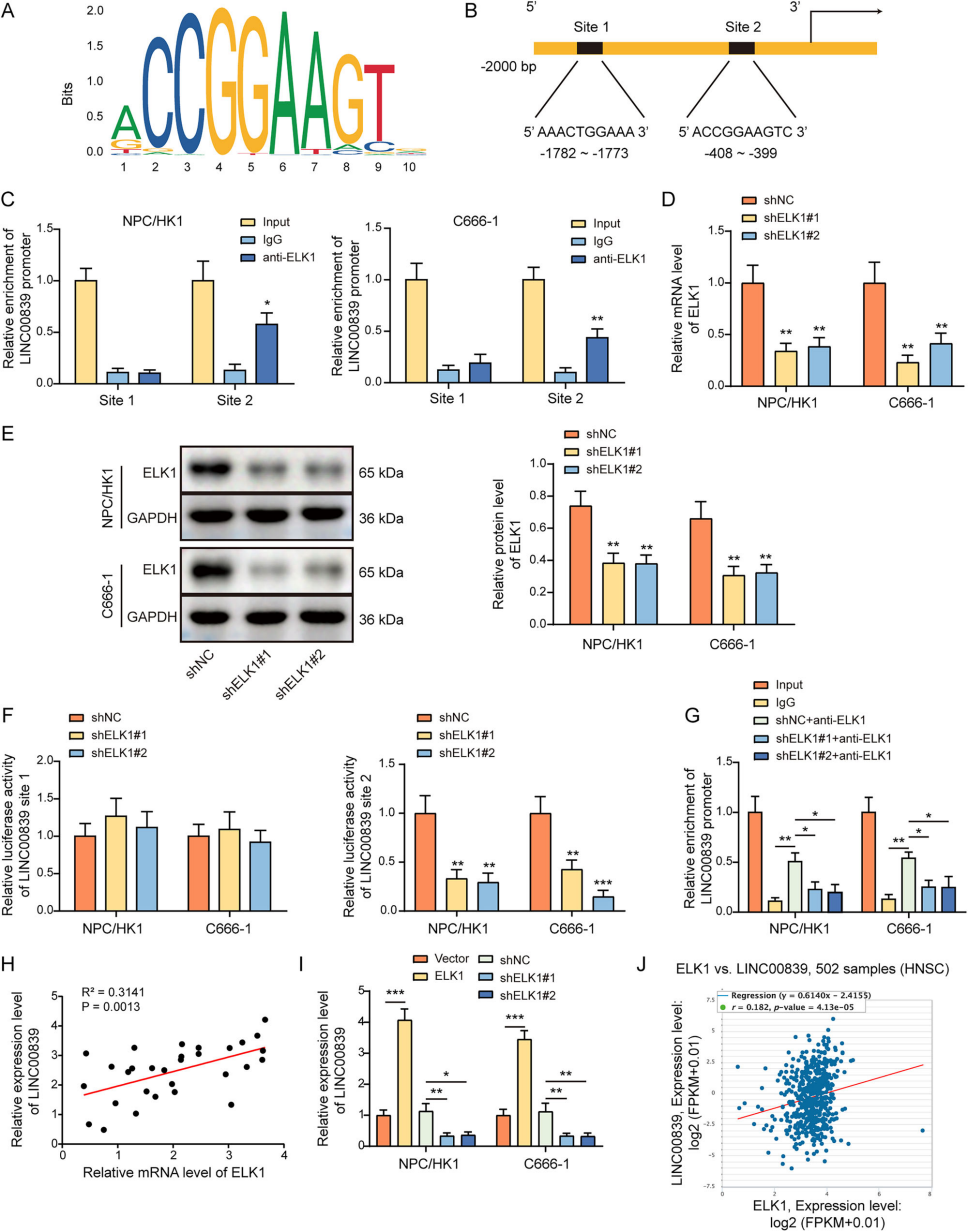
ELK1 directly binds to LINC00839 promoter to activate its transcription. **A** JASPAR database predicted the binding motif of transcription factor ELK1. **B** The putative binding sites of ELK1 to LINC00839 promoter were predicted by JASPAR database. **C** ChIP assay validated the enrichment of binding sites in LINC00839 promoter after immunoprecipitation by ELK1 antibody. **D**, **E** The expression level of ELK1 in NPC cells transfected with shNC or shELK1#1/2 was determined by qRT-PCR and western blotting. **F** The interaction between ELK1 and LINC00839 promoter site 1/2 was determined by dual-luciferase reporter assay in ELK1-depleted NPC cells. **G** The binding of ELK1 to LINC00839 promoter in ELK1-depleted NPC cells was detected by ChIP assay. **H** Pearson correlation analysis of ELK1 and LINC00839 levels in NPC tissues (*n* = 30). **I** qRT-PCR evaluated the expression level of LINC00839 in NPC cells after ELK1 overexpression or knockdown. **J** The correlation between ELK1 and LINC00839 in HNSC was analyzed by ENCORI database (*n* = 502). *P < 0.05, **P < 0.01, and ***P < 0.001.

As a transcription factor, ELK1 modulates the transcription of target genes^31^. We then explored whether ELK1 regulated the transcription and expression of LINC00839 in NPC cells. JASPAR database predicted the binding motifs of transcription factor ELK1 (Fig. 2A), and there were two putative binding sites (site 1 and 2) of ELK1 to LINC00839 promoter (Fig. 2B). ChIP assay showed that LINC00839 promoter site 2, but not site 1, was enriched by ELK1 antibody (Fig. 2C). Subsequently, ELK1 expression was knocked down by specific shELK1#1/2 in NPC/HK1 and C666-1 cells (Fig. 2D, E). The relative luciferase activity of LINC00839 promoter (site 2, rather than site 1) was evidently decreased by ELK1 depletion (Fig. 2F). Besides, ChIP assay showed that ELK1 silencing reduced the enrichment of LINC00839 promoter after immunoprecipitation by ELK1 antibody (Fig. 2G). Notably, Pearson correlation analysis indicated a positive correlation between ELK1 and LINC00839 levels in NPC tissues (Fig. 2H). Additionally, LINC00839 expression was obviously elevated in ELK1-overexpressed cells, while lowered in ELK1-silenced cells (Fig. 2I). ENCORI database further suggested that LINC00839 expression was positively correlated with ELK1 expression in HNSC tissues (Fig. 2J). Taken together, ELK1 directly bound to LINC00839 promoter to trigger its transcription and expression in NPC cells.

### Overexpression of ELK1 or LINC00839 reduces the sensitivity of NPC cells to ferroptosis

Subsequently, we aimed to screen out cell death pathways regulated by ELK1 or LINC00839 using various inhibitors, including Ferrostatin-1 (ferroptosis inhibitor), z-VAD-fmk (apoptosis inhibitor), Chloroquine (autophagy inhibitor), MCC950 (pyroptosis inhibitor), Necrostatin-1 (necroptosis inhibitor). As shown in supplementary Fig. S1A, the reduced cell viability of NPC cells mediated by shELK1#1 or shLINC00839#1 was only counteracted by Ferrostatin-1, but not affected by other inhibitors. Thus, ELK1 or LINC00839 knockdown repressed survival of NPC cells via inducing ferroptosis, rather than other cell death pathways. Next, we aimed to select the optimal transfection time of shLINC00839#1 in combination with Erastin treatment. We found that LINC00839 knockdown remarkably reduced cell viability (Fig. S2A), GSH level (Fig. S2C), GPX4 activity (Fig. S2D), SLC7A11 and GPX4 protein levels (Fig. S2E), while enhanced Fe^2+^ (Fig. S2B) and ACSL4 (Fig. S2E) levels in the presence of Erastin, among which pre-transfection with shLINC00839#1 at 12 h showed most significant changes (Fig. S2A-E). Thus, the NPC cells were pre-transfected with shLINC00839#1 for 12 h and then treated with Erastin for 24 h.

**Fig. 3:**
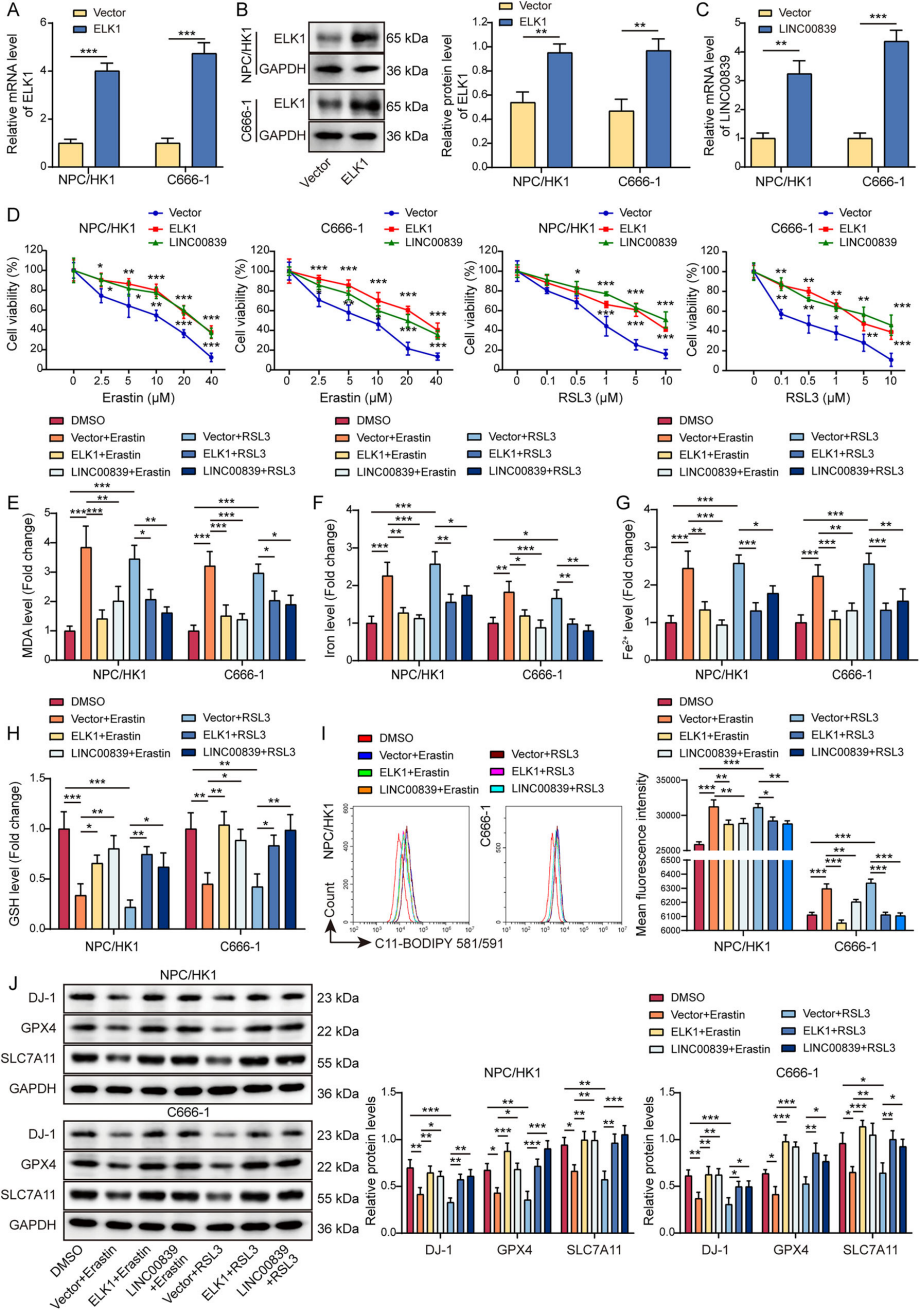
Overexpression of ELK1 or LINC00839 induces ferroptosis resistance of NPC cells. **A**, **B** The expression level of ELK1 in NPC cells transfected with ELK1 overexpression vector or control vector was detected by qRT-PCR and western blotting. **C** The expression level of LINC00839 in NPC cells transfected with LINC00839 overexpression vector or control vector was detected by qRT-PCR. **D** NPC cells transfected with ELK1 or LINC00839 overexpression vector were exposed to various concentrations of Erastin (2.5, 5, 10, 20, 40 μM) or RSL3 (0.1, 0.5, 1, 5, 10 μM) for 24 h. Cell viability was assessed by CCK-8 assay. **E** MDA, **F** iron, **G** Fe^2+^, **H** GSH levels were determined in NPC cells transfected with ELK1 or LINC00839 overexpression vector after treatment with Erastin (10 μM) or RSL3 (1 μM) for 24 h. **I** Lipid ROS level was measured by C11-BODIPY 581/591 probe and flow cytometry. **J** Western blotting analysis of DJ-1, GPX4, and SLC7A11 protein abundance. *P < 0.05, **P < 0.01, and ***P < 0.001.

Ferroptosis is a specific pattern of cell death triggered by lipid peroxidation, and ferroptosis induction represents as an effective strategy for cancer therapy^32^. To investigate the influence of ELK1 or LINC00839 on the sensitivity of NPC cells to ferroptosis inducers Erastin and RSL3, ELK1 or LINC00839 overexpression plasmid was transfected into NPC cells. The overexpression efficiencies of ELK1 (Fig. 3A-B) and LINC00839 (Fig. 3C) were validated. Moreover, the NPC cell viability was dose-dependently reduced by Erastin or RSL3, whereas ELK1/LINC00839 overexpression remarkably reversed this change (Fig. 3D). In addition, Erastin or RSL3 treatment enhanced MDA (Fig. 3E), iron (Fig. 3F), Fe^2+^ (Fig. 3G) levels but reduced GSH level (Fig. 3H) in NPC cells, which could be counteracted by overexpression of ELK1/LINC00839. We also found that lipid ROS level was remarkably elevated by Erastin or RSL3 treatment in NPC cells; however, this change was abolished by ELK1/LINC00839 overexpression (Fig. 3I). It has been reported that DJ-1 could inhibit ferroptosis via activating the Nrf2/GPX4 pathway^33^. SLC7A11, a cystine-glutamate antiporter, has been verified to repress ferroptosis by promoting GSH production^34^. We found that DJ-1, GPX4, and SLC7A11 levels were down-regulated in Erastin or RSL3-treated NPC cells, which were reversed by ELK1/LINC00839 overexpression (Fig. 3J). Thus, our data revealed that ELK1/LINC00839 overexpression was responsible for ferroptosis resistance in NPC cells.

### Overexpression of LINC00839 reverses ELK1 knockdown-induced ferroptosis in NPC cells

**Fig. 4:**
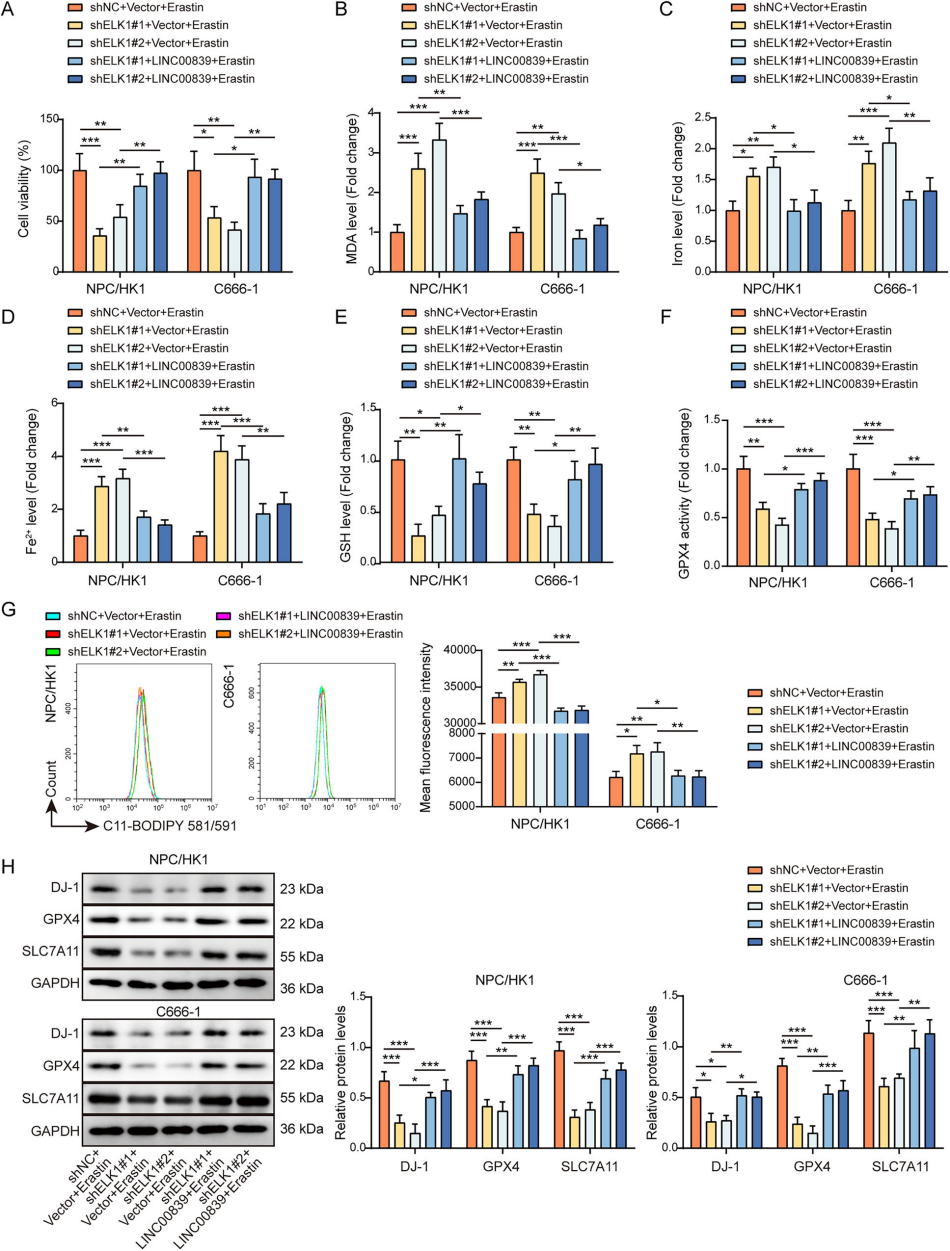
LINC00839 overexpression abolishes ELK1 knockdown-induced NPC cell sensitivity to ferroptosis. NPC cells were transfected with shELK1#1/2 in combination with or without LINC00839 overexpression vector upon Erastin (10 μM) exposure. **A** Cell viability was measured by CCK-8. **B** MDA, **C** iron, **D** Fe^2+^, and **E** GSH levels, and **F** GPX4 activity were detected by commercial kits. **G** C11-BODIPY 581/591 probe and flow cytometry evaluated the lipid ROS level. **H** Protein levels of DJ-1, GPX4, and SLC7A11 were assessed by western blotting. *P < 0.05, **P < 0.01, and ***P < 0.001.

To determine whether ELK1/LINC00839 axis was involved in ferroptosis regulation, Erastin-treated NPC cells were transfected with shELK1#1/2 together with or without LINC00839 overexpression plasmid. NPC cell viability was significantly reduced by ELK1 silencing, which was restored by LINC00839 overexpression (Fig. 4A). Furthermore, silencing of ELK1 significantly raised MDA (Fig. 4B), iron (Fig. 4C), Fe^2+^ (Fig. 4D), and lipid ROS (Fig. 4G) levels, while reduced GSH level (Fig. 4E) and GPX4 activity (Fig. 4F) in NPC cells upon Erastin exposure, which were reversed by LINC00839 overexpression. Accordingly, ELK1 knockdown-mediated down-regulation of DJ-1, GPX4, and SLC7A11 in Erastin-treated NPC cells was counteracted by LINC00839 overexpression (Fig. 4H). In addition, we explored the influence of ELK1/LINC00839 axis on other ferroptosis-related pathways. We found that Nrf2 and HO-1 of antioxidant signaling pathway were down-regulated by ELK1 silencing in Erastin-treated NPC cells, and this change disappeared when LINC00839 was overexpressed, whereas the levels of TFR1 and FTH1 of iron metabolism pathway were not changed (Fig. S3A). Besides, ELK1/LINC00839 was positively correlated with ferroptosis marker GPX4 in HNSC tissues (Fig. S3B). Collectively, ELK1 knockdown sensitized NPC cells to ferroptosis via repressing LINC00839 expression.

### LINC00839 silencing promotes RCHY1-mediated ubiquitination and degradation of DJ-1

**Fig. 5:**
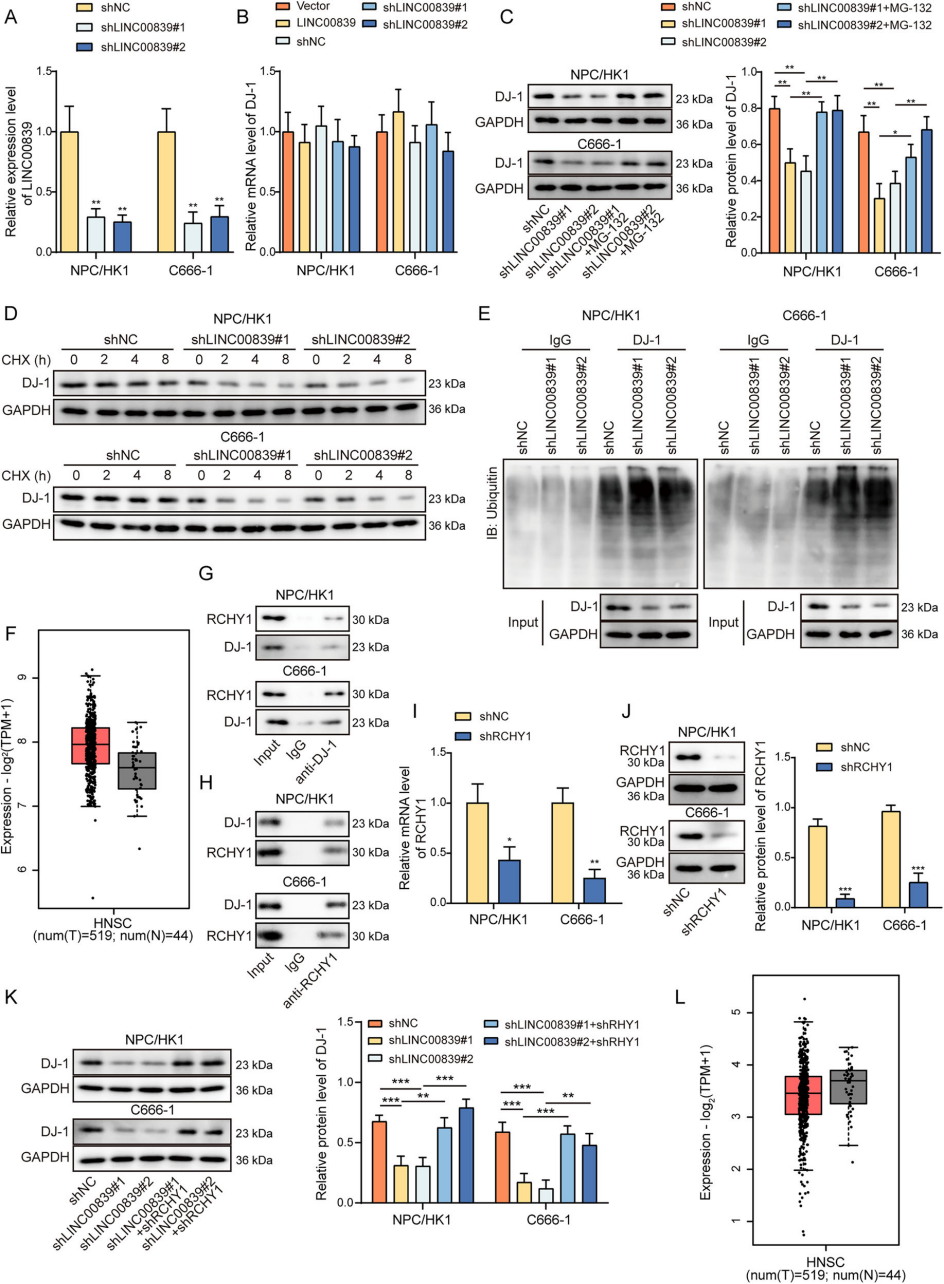
LINC00839 silencing promotes RCHY1-mediated ubiquitination and degradation of DJ-1. **A** NPC cells were transfected with shNC or shLINC00839#1/2. The silencing efficiency of LINC00839 was verified by qRT-PCR. **B** NPC cells were transfected with LINC00839 overexpression vector or shLINC00839#1/2, and the mRNA level of DJ-1 was determined by qRT-PCR. **C** shLINC00839#1/2-transfected NPC cells were treated with or without MG-132 (20 μM), and DJ-1 protein abundance was analyzed by western blotting. **D** NPC cells were exposed to 50 μg/mL CHX for 2, 4, 8 h. The protein abundance of DJ-1 in NPC cells transfected with shNC or shLINC00839#1/2 was detected by western blotting. **E** The ubiquitin level of DJ-1 after silencing of LINC00839 was evaluated by Co-IP. **F** GEPIA database analyzed differential expression of DJ-1 in HNSC tissues (Tumor = 519, Normal = 44). **G**, **H** The interplay between RCHY1 and DJ-1 proteins was validated by Co-IP assay using anti-DJ-1 and anti-RCHY1, respectively. **I**, **J** The knockdown efficiency of RCHY1 was verified by qRT-PCR and western blotting. **K** NPC cells were transfected with shLINC00839#1/2 together with or without shRCHY1. The protein level of DJ-1 was evaluated by western blotting. **L** Differential expression of RCHY1 in HNSC tissues was evaluated by GEPIA database (Tumor = 519, Normal = 44). *P < 0.05, **P < 0.01, and ***P < 0.001.

To further explore the downstream mechanism through which LINC00839 induced ferroptosis resistance, NPC cells were transfected with shLINC00839#1/2. qRT-PCR confirmed the silencing efficiency of LINC00839 (Fig. 5A). In addition, LINC00839 overexpression or silencing did not affect the mRNA expression of DJ-1 (Fig. 5B). Of note, the protein level of DJ-1 was distinctly reduced by LINC00839 silencing in NPC cells, which was abolished after treatment with proteasome inhibitor MG-132 (Fig. 5C). Moreover, CHX-induced degradation of DJ-1 protein was accelerated by LINC00839 depletion in NPC cells (Fig. 5D). Furthermore, the ubiquitin level of DJ-1 protein was remarkably enhanced by LINC00839 silencing (Fig. 5E). GEPIA database indicated that DJ-1 was up-regulated in HNSC tissues (Fig. 5F). Notably, Co-IP assay demonstrated a direct interaction between DJ-1 protein and E3 ligase RCHY1 after immunoprecipitation using anti-DJ-1 (Fig. 5G) and anti-RCHY1 (Fig. 5H), respectively. To investigate whether LINC00839 regulated DJ-1 protein expression via RCHY1, shRCHY1 vector was transfected into NPC cells. Knockdown efficiency of RCHY1 mRNA (Fig. 5I) and protein (Fig. 5J) was validated by qRT-PCR and western blotting. In addition, RCHY1 knockdown effectively reversed LINC00839 silencing-mediated down-regulation of DJ-1 protein (Fig. 5K). As analyzed by GEPIA database, RCHY1 was down-regulated in HNSC tissues (Fig. 5L). Therefore, LINC00839 depletion promoted ubiquitination and degradation of DJ-1 protein with the assistance of RCHY1.

### LINC00839 interacts with UPF1 to reduce RCHY1 mRNA stability and ferroptosis in NPC cells

**Fig. 6:**
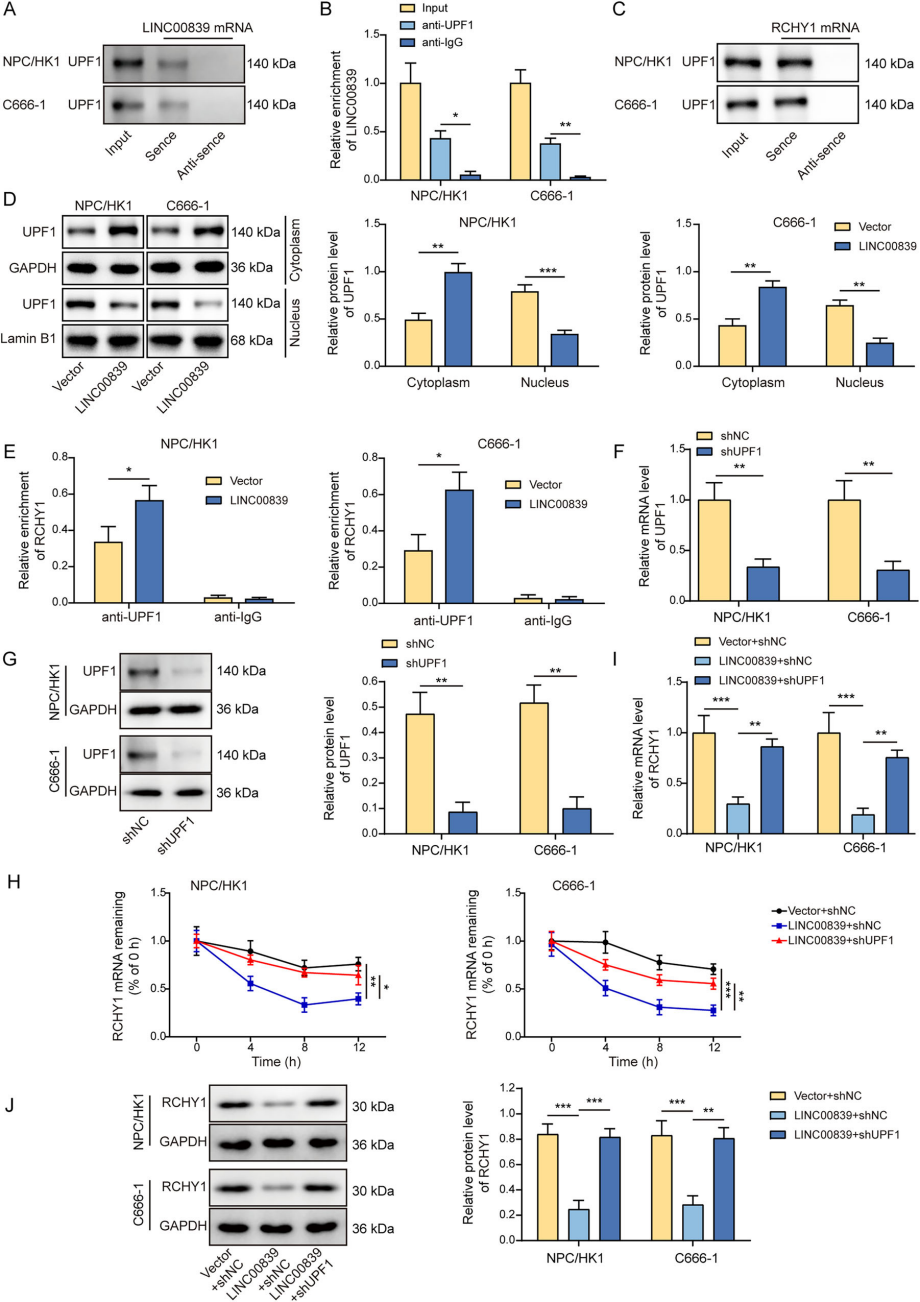
LINC00839 destabilizes RCHY1 mRNA via recruitment of UPF1. **A** The binding of LINC00839 to UPF1 protein was determined by RNA pull-down assay. **B** The interaction between UPF1 protein and LINC00839 was evaluated by RIP assay. **C** The interplay between RCHY1 mRNA and UPF1 protein was evaluated by RNA pull-down assay. **D** The nuclear and cytoplasmic expression of UPF1 protein was assessed by western blotting in LINC00839-overexpressed NPC cells. **E** RIP assay analyzed the binding of UPF1 protein to RCHY1 mRNA in LINC00839-overexpressed NPC cells. **F**, **G** The silencing efficiency of UPF1 was verified by qRT-PCR and western blotting. **H** NPC cells were treated with 2 μg/mL actinomycin D for 4, 8, 12 h, and the stability of RCHY1 mRNA was detected by qRT-PCR. **I**, **J** RCHY1 expression was measured by qRT-PCR and western blotting. *P < 0.05, **P < 0.01, and ***P < 0.001.

Given that RCHY1-mediated ubiquitination of DJ-1 protein was involved in the regulatory mechanism of LINC00839 in ferroptosis resistance, we further explored how LINC00839 modulated RCHY1 expression. RNA pull-down assay indicated that UPF1, an RNA binding protein, could be enriched by LINC00839 probe (Fig. 6A). Furthermore, RIP assay revealed that LINC00839 was immunoprecipitated by UPF1 antibody (Fig. 6B). RNA pull-down assay further proved that RCHY1 mRNA probe remarkably enhanced the enrichment of UPF1 protein (Fig. 6C). Besides, western blotting was conducted to evaluate the influence of LINC00839 on subcellular localization of UPF1. The results indicated that LINC00839 overexpression facilitated the translocation of UPF1 from the nucleus to cytoplasm of NPC cells (Fig. 6D). UPF1-mediated recruitment of RCHY1 mRNA was strengthened by LINC00839 overexpression (Fig. 6E). Transfection with shUPF1 effectively reduced UPF1 expression in NPC cells (Fig. 6F, G). Moreover, actinomycin D-induced degradation of RCHY1 mRNA was promoted by overexpression of LINC00839, whereas UPF1 silencing exerted an opposite role (Fig. 6H). Besides, LINC00839 overexpression-mediated down-regulation of RCHY1 mRNA (Fig. 6I) and protein (Fig. 6J) levels was neutralized by UPF1 knockdown. These results suggested that LINC00839 reduced RCHY1 mRNA stability in NPC cells via recruitment of UPF1.

To verify the regulation of LINC00839/UPF1 axis in ferroptosis, Erastin-treated NPC cells were transfected with LINC00839 overexpression vector combined with or without shUPF1. We found that RCHY1 was down-regulated, while DJ-1, SLC7A11, and GPX4 were up-regulated by LINC00839 overexpression. However, LINC00839 overexpression-mediated these changes were abolished by UPF1 knockdown (Fig. S4A). In addition, LINC00839 overexpression did not affect UPF1 expression (Fig. S4A). Cell viability was enhanced by LINC00839 overexpression, which was reversed by UPF1 depletion (Fig. S4B). Besides, LINC00839 overexpression evidently reduced MDA (Fig. S4C), iron (Fig. S4D), Fe^2+^ (Fig. S4E), and lipid ROS (Fig. S4H) levels, while raised GSH level (Fig. S4F) and GPX4 activity (Fig. S4G) in Erastin-stimulated NPC cells, whereas these alterations were counteracted by UPF1 silencing. Therefore, UPF1 was involved in LINC00839-induced ferroptosis resistance of NPC cells.

### RCHY1 knockdown or DJ-1 overexpression abolishes LINC00839 silencing-induced sensitivity to ferroptosis of NPC cells

**Fig. 7:**
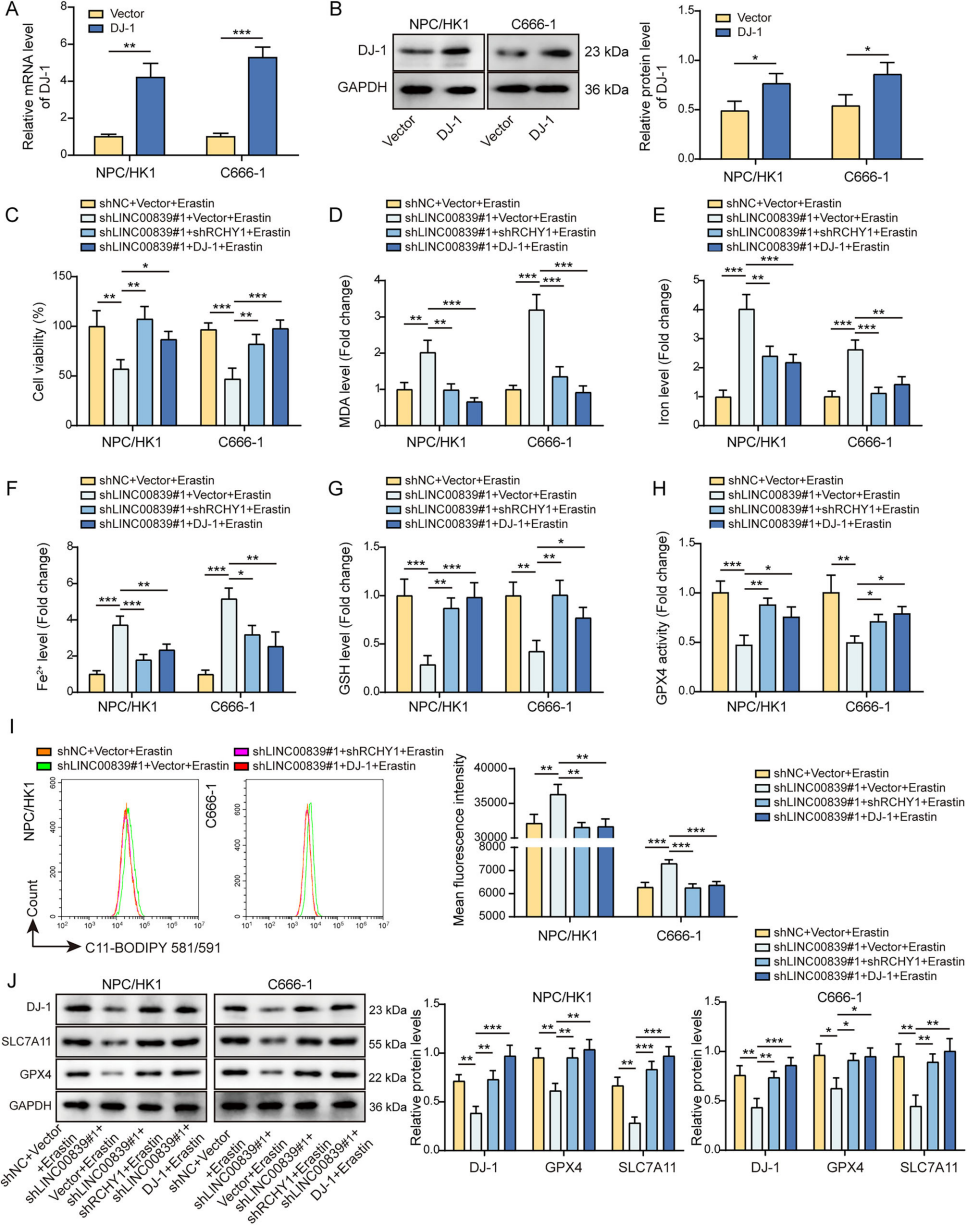
RCHY1 knockdown or DJ-1 overexpression abolishes LINC00839 silencing-induced sensitivity to ferroptosis of NPC cells. **A**, **B** DJ-1 overexpression efficiency was confirmed by qRT-PCR and western blotting. NPC cells were transfected with shLINC00839#1 together with shRCHY1 or DJ-1 overexpression vector upon Erastin (10 μM) exposure. **C** Cell viability was evaluated by CCK-8 assay. **D** MDA, **E** iron, **F** Fe^2+^, and **G** GSH levels, and **H** GPX4 activity were determined by commercial kits. **I** Lipid ROS level was detected by C11-BODIPY 581/591 probe and flow cytometry. **J** Protein abundance of DJ-1, GPX4, and SLC7A11 was detected by western blotting analysis. *P < 0.05, **P < 0.01, and ***P < 0.001.

To verify the involvement of RCHY1/DJ-1 axis in LINC00839-mediated ferroptosis resistance, rescued experiments were conducted. For this purpose, NPC cells were transfected with shLINC00839#1 together with or without shRCHY1/DJ-1 overexpression plasmid in the presence of Erastin. DJ-1 was overexpressed in NPC cells after DJ-1 overexpression plasmid transfection (Fig. 7A, B). Functionally, the reduced cell viability of LINC00839-depleted cells was restored by RCHY1 knockdown or DJ-1 overexpression (Fig. 7C). Additionally, LINC00839 silencing-mediated the increased MDA (Fig. 7D), iron (Fig. 7E), Fe^2+^ (Fig. 7F), and lipid ROS (Fig. 7I) levels, and the decreased GSH content (Fig. 7G) and GPX4 activity (Fig. 7H) were counteracted by co-transfection with shRCHY1 or DJ-1 overexpression plasmid. Besides, RCHY1 silencing or DJ-1 overexpression reversed LINC00839 deficiency-induced down-regulation of DJ-1, SLC7A11, and GPX4 in NPC cells (Fig. 7J). The above observations demonstrated that LINC00839 silencing enhanced ferroptosis sensitivity of NPC cells through modulating the RCHY1/DJ-1 axis.

### Knockdown of ELK1 or LINC00839 suppresses tumor growth through increasing sensitivity to ferroptosis

**Fig. 8:**
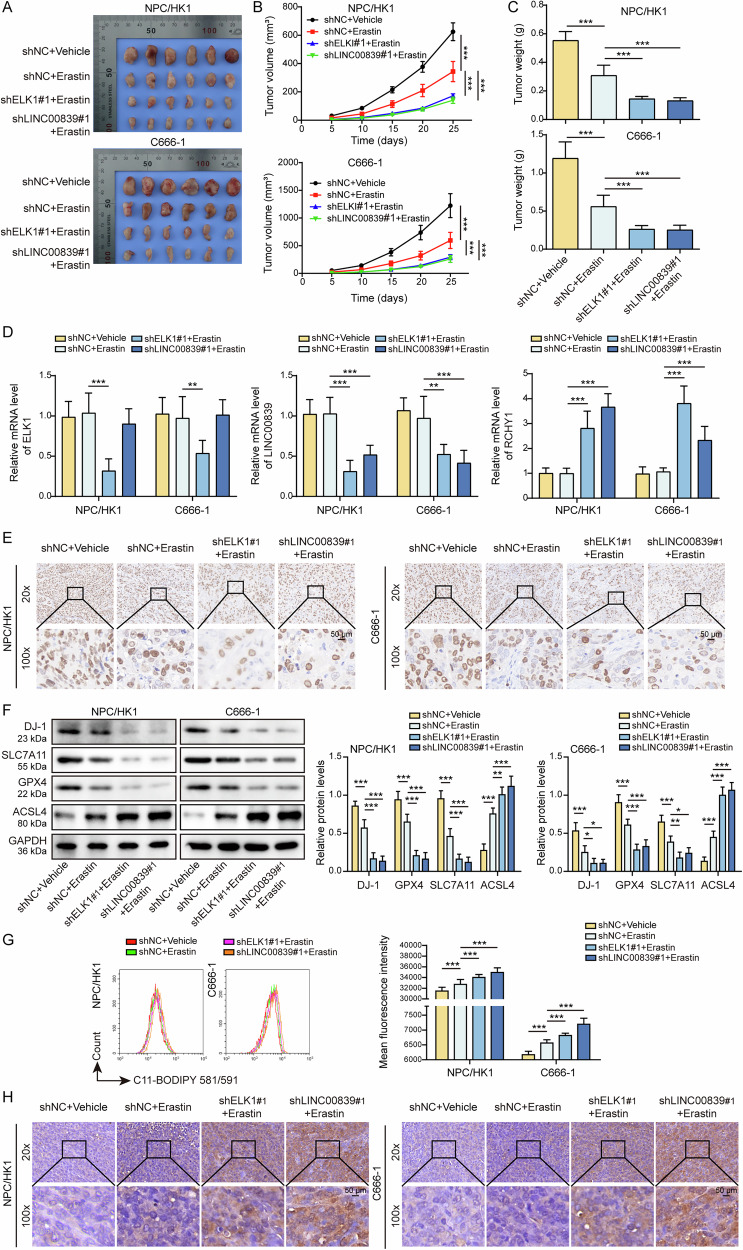
Knockdown of ELK1 or LINC00839 suppresses tumor growth through promoting ferroptosis. NPC/HK1 or C666-1 cells stably infected with lentiviruses carrying shELK1#1, shLINC00839#1, or shNC were subcutaneously injected into BALB/c nude mice. There were four experimental groups (*n* = 6 per group): shNC+Vehicle, shNC+Erastin, shELK1#1+Erastin, shLINC00839#1+Erastin. The mice in Erastin groups were intraperitoneally injected with 15 mg/kg Erastin every other day after 5 days of transplantation. **A** Xenograft tumors of nude mice in each group. **B** Growth curve of xenograft tumors in each group. **C** The tumor weight of xenograft tumors in each group. **D** The expression levels of ELK1, LINC00839, and RCHY1 were assessed by qRT-PCR in xenograft tumor tissues. **E** Ki-67 positive cells were observed by immunohistochemical staining. Scale bar, 50 μm. **F** Protein levels of DJ-1, GPX4, ACSL4, and SLC7A11 in tumors were evaluated by western blotting. **G** Lipid ROS level in tumors was evaluated by C11-BODIPY 581/591 probe and flow cytometry. **H** 4-HNE expression was determined by immunohistochemical staining. Scale bar, 50 μm. *P < 0.05, **P < 0.01, and ***P < 0.001.

Next, we sought to validate the influence of ELK1 or LINC00839 on ferroptosis sensitivity in a xenograft mouse model in vivo. We observed that single treatment with Erastin slightly decreased the volume and weight of xenografts, which could be reinforced by combination with ELK1/LINC00839 silencing (Fig. 8A–C). The expression levels of ELK1, LINC00839, and RCHY1 in xenografts were not affected by Erastin treatment. However, ELK1 silencing inhibited LINC00839 expression and raised RCHY1 expression in xenografts (Fig. 8D). LINC00839 knockdown only increased RCHY1 mRNA abundance, but not affected ELK1 mRNA level in tumors (Fig. 8D). Immunohistochemical staining indicated that there were fewer Ki-67 positive cells in xenograft tumors of Erastin plus ELK1/LINC00839 silencing group as compared with Erastin single treatment group (Fig. 8E). Western blotting results showed that DJ-1, SLC7A11, and GPX4 were down-regulated, while ACSL4 was up-regulated in tumors from Erastin group, which were intensified by deficiency of ELK1 or LINC00839 (Fig. 8F). Additionally, treatment with Erastin raised lipid ROS level in tumor tissues, which was enhanced by ELK1 or LINC00839 silencing (Fig. 8G). Besides, ELK1 or LINC00839 silencing further enhanced 4-HNE expression that was up-regulated by Erastin (Fig. 8H).

**Fig. 9:**
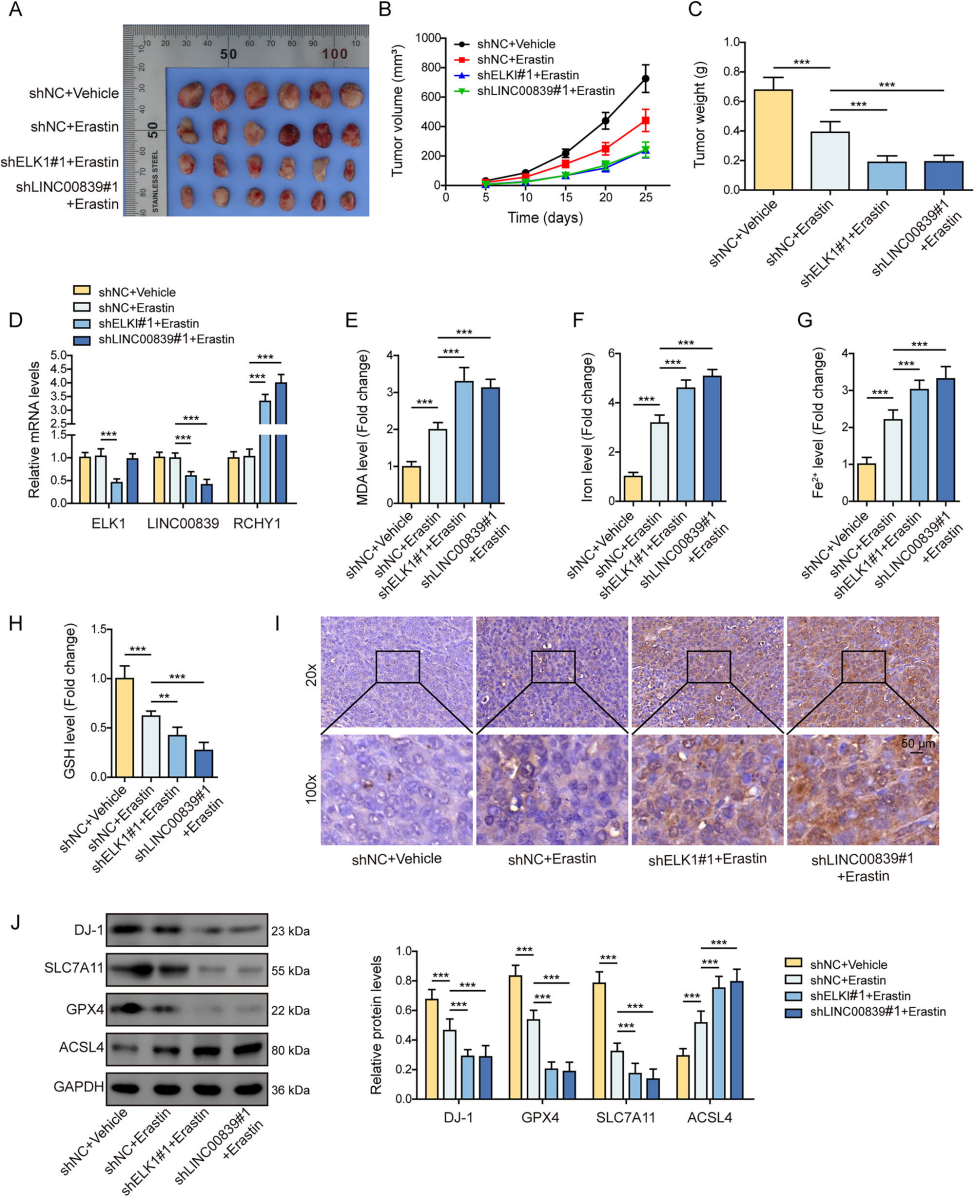
ELK1 or LINC00839 silencing delayed PDX tumor growth by promoting ferroptosis. NPC patient-derived tumor tissue fragments were subcutaneously implanted into the flanks of NCG mice. There were four experimental groups (*n* = 6 per group): shNC+Vehicle, shNC+Erastin, shELK1#1+Erastin, shLINC00839#1+Erastin. Lentiviruses carrying shELK1#1, shLINC00839#1, or shNC were delivered via intratumoral injection on day 5. After 10 days of PDX transplantation, Erastin (15 mg/kg) was administered every other day via intraperitoneal injection. **A** PDX tumors of mice in each group. **B** Growth curve of PDX tumors in each group. **C** The tumor weight of PDX tumors in each group. **D** The expression levels of ELK1, LINC00839, and RCHY1 were analyzed by qRT-PCR in PDX tumor tissues. **E** MDA, **F**iron, **G** Fe^2+^, and **H** GSH levels in PDX tumors were detected. **I** 4-HNE expression was analyzed by immunohistochemical staining. Scale bar, 50 μm. **J** Protein levels of DJ-1, GPX4, ACSL4, and SLC7A11 in PDX tumors were assessed by western blotting. **P < 0.01 and ***P < 0.001.

To validate the above findings, we established patient-derived xenograft (PDX) model in NCG mice. The results showed that tumor volume and weight was moderately decreased by single treatment with Erastin, which was amplified by combination with ELK1 or LINC00839 silencing (Fig. 9A–C). Erastin treatment did not influence the levels of ELK1, LINC00839, and RCHY1 in tumors. ELK1/LINC00839 knockdown up-regulated RCHY1 expression, while LINC00839 did not affect ELK1 level (Fig. 9D). In addition, Erastin treatment enhanced MDA (Fig. 9E), iron (Fig. 9F), Fe^2+^ (Fig. 9G), while lowered GSH (Fig. 9H) level in PDX tumors, which were intensified by ELK1 or LINC00839 depletion. Furthermore, DJ-1, SLC7A11, and GPX4 levels were declined, but ACSL4 and 4-HNE levels were elevated in Erastin-treated PDX tumors, and these alterations were more obvious after ELK1 or LINC00839 deficiency (Fig. 9I, J). Collectively, our data supported that ELK1 or LINC00839 silencing enhanced ferroptosis sensitivity to delay NPC growth in vivo.

### Deficiency of ELK1 or LINC00839 enhanced anti-cancer efficiency of Sorafenib by boosting ferroptosis

**Fig. 10:**
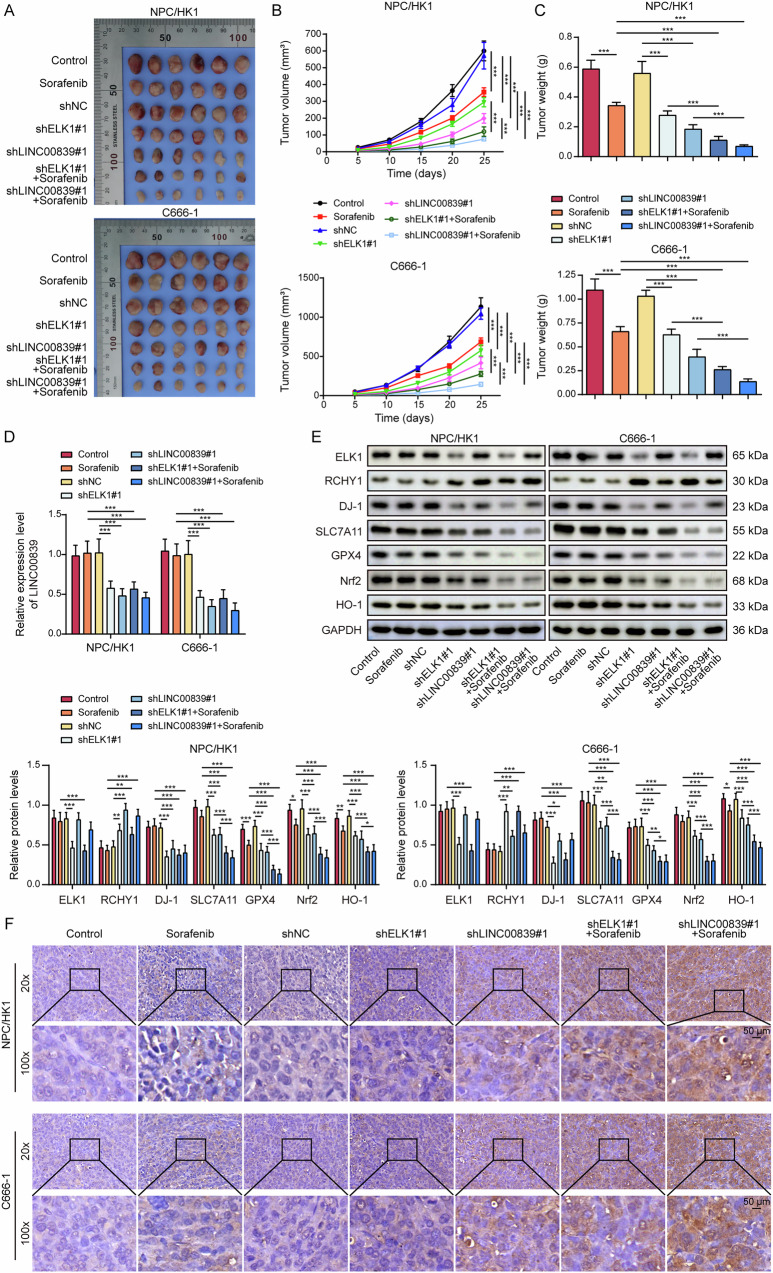
Loss of ELK1 or LINC00839 enhanced anti-cancer efficiency of Sorafenib by boosting ferroptosis. NPC/HK1 or C666-1 cells stably infected with lentiviruses carrying shELK1#1, shLINC00839#1, or shNC were subcutaneously injected into BALB/c nude mice. There were 7 experimental groups (*n* = 6 per group): control, Sorafenib, shNC, shELK#1, shLINC00839#1, shELK#1+Sorafenib, shLINC00839#1+Sorafenib. The mice in Sorafenib groups were intraperitoneally injected with 10 mg/kg Sorafenib every other day after 5 days of transplantation. **A** Xenograft tumors of nude mice in each group. **B** Growth curve of xenograft tumors in each group. **C** The tumor weight of xenograft tumors in each group. **D** LINC00839 expression in xenograft tumor tissues was measured by qRT-PCR. **E** Protein levels of ELK1, RCHY1, DJ-1, SLC7A11, GPX4, Nrf2, and HO-1 in tumors were evaluated by western blotting. **F** 4-HNE expression was detected by immunohistochemical staining. Scale bar, 50 μm. *P < 0.05, **P < 0.01, and ***P < 0.001.

Finally, we investigated the influence of ELK1/LINC00839 in combination with Sorafenib (a ferroptosis-inducing chemotherapeutic drug) on tumor growth in vivo. We found that Sorafenib single treatment slightly reduced tumor volume and weight, which was reinforced by combination with ELK1 or LINC00839 knockdown (Fig. 10A–C). LINC00839 expression in tumor tissues was not influenced by Sorafenib treatment; however, ELK1 or LINC00839 silencing strikingly repressed LINC00839 expression (Fig. 10D). Additionally, ELK1, RCHY1, and DJ-1 protein levels were not regulated by Sorafenib single treatment, and depletion of ELK1/LINC00839 enhanced RCHY1, but reduced DJ-1 protein abundance in the presence or absence of Sorafenib (Fig. 10E). Sorafenib-mediated regulation of SLC7A11, GPX4, Nrf2, HO-1 (Fig. 10E), and 4-HNE (Fig. 10F) expression was further intensified by deficiency of ELK1/LINC00839. To sum up, depletion of ELK1/LINC00839 strengthened the anti-cancer action of Sorafenib in NPC.

## Discussion

Ferroptosis participates in the therapeutic response of different human malignancies, including NPC^35^. Biomarkers are pivotal for translating ferroptosis research into clinical practice, since they can identify patients most likely to benefit from ferroptosis-targeted therapies. In addition, both chemotherapy and radiotherapy exert therapeutic actions through triggering ferroptosis of cancer cells^36^. However, ferroptosis resistance is a key driver for chemoradiotherapy resistance in clinical application. Currently, about 15–20% of NPC patients still suffer local recurrence or distant metastasis because of chemoradiotherapy resistance^37^. Thus, overcoming ferroptosis resistance remains an unmet clinical need, and uncovering underlying mechanism of ferroptosis resistance has been identified as a novel approach to improve chemoradiotherapy efficiency of NPC patients.

**Fig. 11:**
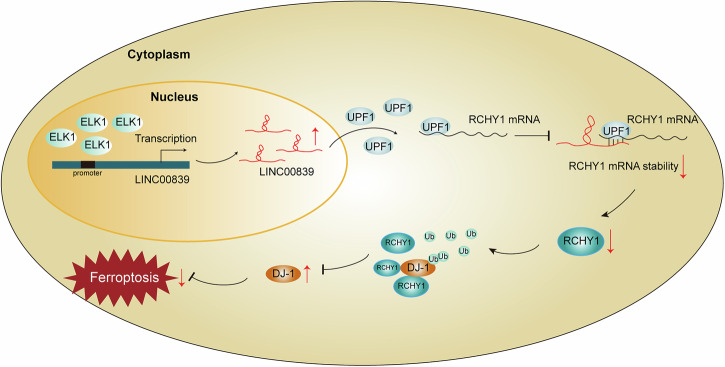
The potential mechanism of LINC00839 inhibiting ferroptosis in NPC. ELK1 binds to LINC00839 promoter and promotes its transcription. LINC00839 binds to UPF1 and lowers the mRNA stability of RCHY1. Downregulation of RCHY1 represses DJ-1 ubiquitination and degradation, leading to NPC cell resistance to ferroptosis.

In this study, we discovered that ELK1 bound to LINC00839 promoter to promote its transcription, and subsequently interacted with UPF1 to lower RCHY1 mRNA stability. Furthermore, low RCHY1 expression repressed the ubiquitination and degradation of DJ-1 protein, thereby leading to NPC cell resistance to ferroptosis (Fig. 11). These observations elucidated the potential mechanism of ferroptosis resistance in NPC cells, suggesting elevated expression of ELK1 and LINC00839 may indicate a higher risk of chemoradiotherapy resistance and could serve as biomarkers for stratified treatment in NPC patients. Our findings also identified ELK1/LINC00839 axis as promising treatment targets for NPC patients with chemoradiotherapy resistance. Here, we summarized the novelties of this study as follow: First, we for the first time demonstrated that ELK1 bound to LINC00839 promoter to facilitate its transcription and expression. Second, we provided first evidence for the promotive role of ELK1/LINC00839 axis in ferroptosis resistance in NPC. Third, we firstly reported the decreased expression of RCHY1 in NPC and its promotion to ferroptosis resistance. Fourth, we firstly discovered that LINC00839 recruited UPF1 to reduce RCHY1 mRNA stability. Lastly, we offered first evidence that the decreased RCHY1 expression restrained the ubiquitination and degradation of DJ-1 protein in NPC cells.

ELK1 belongs to the ETS oncogene family, which plays a crucial role in tumorigenesis. For instance, ELK1 protein level was elevated in lung adenocarcinoma, which accelerated lung adenocarcinoma cell metastasis via facilitating epithelial-to-mesenchymal transition^38^. In breast cancer, ELK1 was shown to drive malignant progression by transcriptional activation of KIF26B^39^. Another study showed that ELK1 facilitated the transcription and expression of its target oncogene c-Fos, thus promoting bladder cancer cell growth^40^. It’s worth noting that ELK1 restrained ferroptosis via transcriptional activation of GPX4 during endometrial carcinoma development^30^. However, the influence of ELK1 on ferroptosis of NPC cells has not been explored. In this study, we discovered higher level of ELK1 in NPC tissues as compared with rhinitis samples. Overexpression of ELK1 weakened Erastin or RSL3-induced ferroptosis of NPC cells, whereas ELK1 knockdown strengthened Erastin-mediated inhibition in tumor growth in vitro and in vivo via triggering ferroptosis. Therefore, our observations uncover the promotive role of ELK1 in ferroptosis resistance, thereby weakening the therapeutic efficiency of NPC.

It has been shown that differentially expressed lncRNAs have a close association with the development of NPC^18^. LncRNAs have been identified as effective therapeutic targets for NPC^41^. Mounting evidence revealed that LINC00839 functioned as an oncogene for liver cancer^42^, colorectal cancer^26^, gastric cancer^25^, and so on. Zhang et al. reported that LINC00839 favored the malignant capacities of NPC cells by sponging miR-454-3p to up-regulate c-Met^43^. However, whether LINC00839 takes part in the regulation of ferroptosis resistance during NPC progression remains largely unknown. Interestingly, there was a positive correlation between LINC00839 and ELK1 levels in NPC tissues. In this study, ELK1 served as an upstream regulator of LINC00839, which activated the transcription and expression of LINC00839, thus promoting ferroptosis resistance of NPC cells. It has been confirmed that the glutathione system, antioxidant Nrf2/HO-1 pathway, and iron metabolism pathway are the main regulatory mechanisms of ferroptosis^44^. DJ-1 and Nrf2 have potent antioxidant capacities. For example, activation of the DJ-1/Nrf2 pathway mitigated lipid peroxidation and ferroptosis^45^. In addition, activation of the Nrf2/HO-1 pathway in turn prevented ferroptosis by promoting the transcription of downstream anti-ferroptosis markers, such as GPX4 and SLC7A11^46,47^. Our findings indicated that inhibition of the ELK1/LINC00839 axis induced ferroptosis of NPC cells via inactivation of the Nrf2/HO-1 antioxidant pathway, whereas iron metabolism pathway markers, including TFR1 and FTH1 were not affected. This study provided first evidence that ELK1-mediated transcriptional activation of LINC00839 exerted a promotive role in NPC progression through repressing ferroptosis.

LncRNAs can interact with DNAs, mRNAs, or proteins to regulate gene function and cellular signal transduction at multiple levels^48^. Especially, lncRNAs can post-transcriptionally modulate gene expression via interplaying with RNA-binding proteins to regulate the stability of mRNAs. For example, lncRNA VIM‑AS1 facilitated prostate cancer growth and androgen therapy resistance by enhancing stability of HMGCS1 mRNA via interaction with IGF2BP2^49^. UPF1 is an RNA-binding protein that involved in RNA decay^50^. So far, a series of studies reported that lncRNA/UPF1 axis took part in diverse disorders such as viral myocarditis^51^, Alzheimer’s disease^52^, pulmonary ischemia/reperfusion injury^53^, intracerebral haemorrhage^54^, and so on^55,56^. LncRNA/UPF1 axis also affected multiple biological functions such as autophagic cell death^57^, cancer stem cell-like properties^58^, osteoblast differentiation^56^, and so on^59–61^. However, limited studies reported the influence of lncRNA/UPF1 axis on ferroptosis. In addition, no reports have discovered the upstream transcription factors for lncRNA/UPF1 axis. In this work, we for the first time reported that transcription factor ELK1 promoted LINC00839 transcription, which interacted with UPF1 to drive ferroptosis resistance of NPC cells.

RCHY1 is an E3 ubiquitin ligase that promotes the ubiquitination and degradation of its target proteins^62^. RCHY1-mediated degradation of various proteins exerted multiple biological functions, including DNA damage, cell cycle, and tumorigenesis^63^. Choi et al. reported that RCHY1 inhibited HDAC2 protein expression via the ubiquitin-proteasome pathway in various types of cancers^62^. So far, the influence of RCHY1 on ferroptosis during NPC progression has not been clarified. DJ-1 was documented to restrain ferroptosis through activation of the Nrf2/GPX4 pathway in preeclampsia^33^. Additionally, DJ-1 enhanced the metastatic capacity of tumor cells during the progression of NPC^12^. Although DJ-1 was recognized as an inhibitor of ferroptosis, its influence on NPC cell ferroptosis remains unclear. Up to now, lncRNA/DJ-1 axis has not been reported. In this study, we firstly found that RCHY1 directly interacted with DJ-1 to facilitate ubiquitination-mediated DJ-1 protein degradation. Collectively, our observations uncovered a novel modulatory mechanism of LINC00839 in NPC cell ferroptosis resistance: LINC00839 inhibited ubiquitination and degradation of DJ-1 by post-transcriptional modulation of RCHY1 mRNA.

Ferroptosis resistance has been suggested to be a driver of chemotherapy resistance in NPC. As an anti-cancer drug for NPC, Sorafenib has been shown to induce ferroptosis^64^, but often develops resistance during treatment, thus posing a critical issue. For example, high expression of NAT10 reduced the therapeutic efficacy of Sorafenib via inhibiting ferroptosis in NPC cells^65^. Consistent with this study, we discovered that up-regulation of ELK1 and LINC00839 in NPC reduced the efficiency of Sorafenib via repressing ferroptosis. Knockdown of ELK1 or LINC00839 enhanced ferroptosis sensitivity of NPC cells, thereby promoting the anti-cancer action of Sorafenib. Therefore, our study suggests that ELK1/LINC00839 axis might serve as targets for enhancing Sorafenib chemosensitivity, which offers a new therapeutic option for NPC patients with chemotherapy resistance, particularly those with high expression levels of ELK1 and LINC00839. Furthermore, our observations showed that high expression of ELK1/LINC00839 indicated a lower survival of NPC patients. Therefore, ELK1/LINC00839 up-regulation suggests a high risk of chemotherapy resistance, and might be potential biomarkers for NPC, which provides theoretical foundation for personalized treatment.

Although targeting the ELK1/LINC00839 axis faces several clinical challenges, such as pharmacodynamics, delivery feasibility, and potential off-target effects, we conducted discussions based on a series of experiments and extensive literature review. For pharmacodynamics, based on our experimental findings, a clinical strategy combining LINC00839 inhibition with ferroptosis induction would benefit from a sequential administration schedule. Specifically, pretreatment with LINC00839-targeting intervention for 12 h prior to the ferroptosis-inducing agents (e.g., Erastin or Sorafenib) yielded the most pronounced therapeutic effect. This optimal time window likely facilitates maximal sensitization of tumor cells to ferroptosis, providing a rationale for designing timed combination regimens in future clinical applications. To ensure both efficiency and safety of the delivery system, clinically mature platforms such as lipid nanoparticles (LNP) and GalNAc-conjugated polymers are preferred over laboratory-level non-specific delivery systems (e.g., the cationic liposome transfection reagent Lipofectamine), which exhibit high in vivo toxicity. Notably, LNP has been successfully employed as the delivery system for Patisiran, an FDA-approved siRNA therapeutic. Additionally, incorporating modifications targeting receptors highly expressed in NPC (e.g., EGFR antibody conjugates) may further enhance tumor specificity and minimize off-target effects. As basic research, we are confident that our findings can provide foundation for future clinical trials. Also, the present study has several limitations. First, we found that ELK1/LINC00839 axis did not affect the pyroptosis of NPC cells, which is another type of programmed cell death mediated by inflammasomes. Whether ELK1/LINC00839 axis can regulate other inflammatory pathways deserves to be investigated in the future study. Second, due to the ethical requirements of the mouse tumor model, the tumor weight and volume cannot exceed the limit. Before reaching this limit, the mice need to be euthanized. Therefore, we could not continuously observe the survival rates of mice. In principle, if tumor growth exceeds a certain threshold, it may lead to death via nutrient depletion, organ compression, functional impairment, or metabolic disorders. Third, this study only used two cell lines at the cellular level and RNA-seq validation across patient cohorts was not conducted due to limitations in time and financial resources, but we minimized the influence of tumor tissue heterogeneity by focusing on the following aspects: (1) We analyzed differentially expressed lncRNAs in NPC tissues using three clinical microarray data from GEO database. (2) We employed a series of publicly available bioinformatics databases about HNSC. (3) We collected multiple clinical NPC samples for analysis and verification. (4) We conducted xenograft mouse model and PDX model to validate our conclusion. Therefore, although two cell lines were adopted, we proved our conclusion from multidimensional perspectives.

Taken together, our study demonstrated that ELK1-mediated transcriptional activation of LINC00839 promoted ferroptosis resistance of NPC cells via UPF1-mediated destabilization of RCHY1 mRNA, and subsequent repressing ubiquitination and degradation of DJ-1, thus favoring NPC progression. These findings suggest that targeting ELK1/LINC00829 axis might be an effective therapy to fight against ferroptosis resistance in chemoradiotherapy of NPC patients.

## Methods

### Clinical samples

Rhinitis samples (*n* = 18) and NPC samples (*n* = 30) were obtained at Hunan Provincial Cancer Hospital (Changsha, China). The tissue samples were stored in liquid nitrogen before detection. All participators signed their written consent before our study. The experimental procedures were in accordance with the Declaration of Helsinki and approved by the Independent Ethical Committee of Institute of Clinical Pharmacology, Central South University (CTXY-140007-2).

### Bioinformatics analysis

Three datasets (GSE12452, GSE227541, and GSE118719) containing the lncRNA expression profiles of control and NPC tissues were collected from the Gene Expression Omnibus (GEO) database (https://www.ncbi.nlm.nih.gov/geo/). The differentially expressed lncRNAs in NPC samples were shown by volcano plots, venn diagram, and heat map.

GEPIA database (http://gepia2.cancer-pku.cn/#index) was used to analyze the differential expression of LINC00839, ELK1, DJ-1, and RCHY1 in HNSC. The correlation between LINC00839 and ELK1, as well as the correlation between ELK1/LINC00839 and ferroptosis marker GPX4 in HNSC, was evaluated by ENCORI database (https://rnasysu.com/encori/).

### Cell culture and treatment

Human NPC cell lines, including NPC/HK1, C666-1, 5-8 F, HNE-3, and nasopharyngeal epithelial cell line NP69 were obtained from the iCell Bioscience (Shanghai, China). NP69 cells were cultured with K-SFM medium (Invitrogen, Carlsbad, CA, USA) and the other cells were cultured with RPMI 1640 medium (Invitrogen) containing 10% fetal bovine serum (FBS, Gibco, Grand Island, NY, USA) at 37 °C with 5% CO_2_. To screen out cell death pathways, NPC cells were treated with Ferrostatin-1 (ferroptosis inhibitor, 10 μM, Selleck, Shanghai, China), z-VAD-fmk (apoptosis inhibitor, 10 μM, Selleck), Chloroquine (autophagy inhibitor, 50 μM, Selleck), MCC950 (pyroptosis inhibitor, 30 μM, Selleck), or Necrostatin-1 (necroptosis inhibitor, 40 μM, Selleck) for 24 h. To induce ferroptosis, NPC cells were treated with ferroptosis inducer Erastin (2.5, 5, 10, 20, 40 μM, Selleck) or RSL3 (0.1, 0.5, 1, 5, 10 μM, Selleck) for 24 h.

### Cell transfection

Overexpression plasmids for ELK1, LINC00839, and DJ-1, shRNAs including shELK1#1/2, shLINC00839#1/2, shRCHY1, shUPF1, and negative control shRNA (shNC) were all synthesized from GenePharma (Shanghai, China). NPC/HK1 and C666-1 cells were transiently transfected with these plasmids or shRNAs using Lipofectamine 3000 (Thermo Fisher Scientific, Waltham, MA, USA).

Lentiviruses carrying shELK1#1, shLINC00839#1, or shNC were obtained from GenePharma as well. NPC cells were infected with the above lentiviruses at a multiplicity of infection (MOI) of 30. The stably infected NPC cells were selected using 2 μg/mL puromycin (MedChemExpress, Monmouth Junction, NJ, USA).

### Cell counting kit-8 (CCK-8)

CCK-8 was used to determine cell viability. Briefly, 1 × 10^3^ NPC cells were planted into the 96-well plates. After adding with 10 μL of CCK-8 reagent (BioGLP, San Diego, CA, USA) and incubation for 2 h, the absorbance at 450 nm was detected on a microplate reader.

### Analysis of lipid ROS level

After various treatments, NPC cells were collected and stained with the C11-BODIPY 581/591 probe (50 μM, D3861, Thermo Fisher Scientific) for 2 h away from light. Then, cells were washed with D-Hanks for three times, and the lipid ROS level was determined by flow cytometry (Beckman Coulter, Brea, CA, USA). For the detection of tumor tissues, the tumors were cut into 1 mm^3^ small pieces, followed by enzyme digestion for 30 min at 37 °C. The single-cell suspension was stained with C11-BODIPY 581/591 probe and the lipid ROS level was tested, just like the operational process at the cellular level.

### Detection of malondialdehyde (MDA), iron, and glutathione (GSH) levels

For the determination of ferroptosis, MDA, iron amount (Fe^2+^ and Fe^3+^), and GSH levels were determined using the commercial MDA Content Assay Kit (BC0020, Solarbio, Beijing, China), Iron Assay Kit (ab83366, Abcam, Cambridge, UK), and GSH Content Assay Kit (BC1170, Solarbio), respectively.

### Chromatin immunoprecipitation (ChIP)

The binding of ELK1 to LINC00839 promoter was validated by ChIP using the ChIP Kit (Bes5001, BersinBio, Guangzhou, China). Briefly, NPC cells (1 × 10^7^) were treated with 1% formaldehyde for 10 min at 37 °C for crosslinking, followed by sonication for chromatin fragmentation. The fragmented chromatin was then immunoprecipitated with anti-ELK1 (#51398, Cell Signaling Technology, Danvers, MA, USA) or anti-IgG (ab172730, Abcam) antibody at 4 °C overnight, followed by incubation with the protein A/G beads for 1 h with rotation. After washing with elution buffer at 65 °C for 15 min, the cross-linked DNAs were uncoupled and immunoprecipitated by ethanol. The relative enrichment of LINC00839 promoter in the immunoprecipitated DNAs was evaluated by qPCR.

### Dual-luciferase reporter assay

The sequences of the potential binding site 1/2 of ELK1 to LINC00839 promoter were inserted into the pGL3 vector (Promega, Madison, WI, USA) to establish luciferase reporter vectors. NPC/HK1 and C666-1 cells were co-transfected with the constructed luciferase reporter vectors, together with pRL-TK (Promega), shELK1#1/2, or shNC using Lipofectamine 3000. At 48 h after the transfection, the relative luciferase activity was determined using the Dual-Luciferase Reporter Assay Kit (E1910, Promega).

### Co-immunoprecipitation (Co-IP) assay

Cell lysis was prepared using the Cell lysis buffer for Western and IP (Beyotime, Shanghai, China) containing the protease inhibitor cocktail. Briefly, 500 μL of cell lysates (protein concentration: 1 mg/mL) were immunoprecipitated with anti-DJ-1 (ab76008, Abcam), anti-RCHY1 (ab189907, Abcam), or normal anti-IgG (ab172730, Abcam) antibody at 4 °C overnight, followed by incubation with the protein A/G agarose beads for 2 h with rotation. After washing three times with lysis buffer, the immunoprecipitated proteins were detected using western blotting.

### RNA pull-down assay

RNA pull-down assay was conducted to validate the binding of LINC00839 or RCHY1 mRNA to UPF1 protein using the RNA Pull-Down Kit (Bes5102, BersinBio). In short, the streptavidin magnetic beads were pre-coated with biotinylated LINC00839 or RCHY1 mRNA probe or anti-sense probe for 30 min. Subsequently, the cell lysates were hybridized with probe-coated beads at 4 °C overnight. Finally, the protein level of UPF1 in the immunoprecipitated proteins was analyzed by western blotting.

### RNA immunoprecipitation (RIP)

RIP assay was performed using the RIP Kit (Bes5101, BersinBio). Briefly, NPC cells were lysed with the RIP lysis buffer. The cell lysates were immunoprecipitated with the protein A/G magnetic beads conjugated with anti-UPF1 (#9435, Cell Signaling Technology) or anti-IgG (ab172730, Abcam) antibody for 2 h at 4 °C. The immunoprecipitated RNAs in the beads were purified and the enrichment of LINC00839 or RCHY1 mRNA was assessed by qRT-PCR.

### Detection of RCHY1 mRNA stability

NPC/HK1 and C666-1 cells were seeded into the 24-well plates (5 × 10^5^/well), and then exposed to 2 μg/mL actinomycin D (Selleck) for 0, 4, 8, or 12 h. After the isolation of total RNA, the RCHY1 mRNA level was measured by qRT-PCR.

### GPX4 activity measurement

GPX4 activity in NPC cells was assessed by a commercial GPX4 Activity Assay Kit (E-BC-K883-M, Elabscience, Wuhan, China) following the manufacturer’s protocol.

### Animal models

Male BALB/c nude mice (aged 4–6 weeks) were purchased from the SLAC Laboratory Animal Center (Shanghai, China). Male NOD/ShiLtJGpt-*Prkdc*^em26Cd52^ *Il2rg*^em26Cd22^/Gpt (NCG) mice (aged 6-8 weeks) were purchased from GemPharmatech (Nanjing, China). All mice were maintained under specific-pathogen-free (SPF) conditions, with temperature controlled at 21–23 °C, relative humidity at 40–70%, and a 12-h light/dark cycle. Six mice were included in each group.

For xenograft mouse models, there were two batches of animal experiments. One batch of experiments included 4 groups: shNC+Vehicle, shNC+Erastin, shELK1#1+Erastin, shLINC00839#1+Erastin. The other batch of experiments included 7 groups: control, Sorafenib, shNC, shELK#1, shLINC00839#1, shELK#1+Sorafenib, shLINC00839#1+Sorafenib. NPC/HK1 or C666-1 cells stably infected with lentiviruses carrying shELK1#1, shLINC00839#1, or shNC were subcutaneously injected into the right axillary region of the nude mice. After 5 days of transplantation, the mice in Erastin or Sorafenib groups were intraperitoneally injected with 15 mg/kg Erastin or 10 mg/kg Sorafenib (MedChemExpress) every other day^65^.

For patient-derived xenograft (PDX) model, the NCG mice were divided into four groups: shNC+Vehicle, shNC+Erastin, shELK1#1+Erastin, shLINC00839#1+Erastin. NPC patient-derived tumor tissue fragments with a volume of 1–3 mm^3^ were subcutaneously implanted into the flanks of NCG mice. Lentiviruses carrying shELK1#1, shLINC00839#1, or shNC were delivered via intratumoral injection on day 5. After 10 days of PDX transplantation, Erastin (15 mg/kg) was administered every other day via intraperitoneal injection.

Our study was in accordance with the 3R principles (Reduction, Refinement, Replacement) of animal ethics. All animal experiment procedures and methods were reported in accordance with ARRIVE guidelines. The tumor size was detected using a digital caliper and tumor volume was calculated using the formula: (length × width^2^)/2. Mice were anesthetized by intraperitoneal injection of pentobarbital sodium (50 mg/kg) to induce general anesthesia. All mice were euthanized by cervical dislocation at 25 days after transplantation. All experiments were approved by the Ethics Committee of Changsha Medical University (No.2023051).

### Immunohistochemical staining

The tumor tissues were embedded in paraffin and sectioned into slices. After deparaffinization and rehydration, the slices were subjected to antigen retrieval by 15 mmol/L citrate buffer heating in microwave oven. Subsequently, the slices were blocked with 3% BSA solution and incubated with Ki-67 (1:2000, 27309-1-AP, Proteintech, Wuhan, China) or 4-HNE (1:50, ab48506, Abcam) antibody at 4 °C overnight, followed by incubation with the secondary antibody (1:1000, ab6721/ab97040, Abcam) for 1 h. The slices were then counterstained with DAB working buffer (Solarbio).

### Quantitative reverse transcription polymerase chain reaction (qRT-PCR)

Total RNA was extracted from NPC cells and tissues using the RNAiso Plus reagent (Takara, Tokyo, Japan), and subsequently reverse-transcribed into cDNA using the PrimeScript RT Master Mix (Takara). Real-time PCR was performed with the SYBR Premix ExTaq (Takara). The relative gene levels normalized to GAPDH were calculated using the 2^−ΔΔCt^ method. The primer sequences were listed in Table S1.

### Western blotting

The NPC cells or tissues were lysed with the RIPA reagent (Beyotime) to extract total protein. The Nuclear and Cytoplasmic Protein Extraction Kit (P0028, Beyotime) was used to separate nuclear and cytoplasmic protein samples. Protein concentration was assessed using the Enhanced BCA Protein Assay Kit (Beyotime). The isolated protein samples were separated by sodium dodecyl sulfate-polyacrylamide gel electrophoresis and blotted onto the polyvinylidene fluoride membranes, followed by blocking in 5% skim milk on a shaker for 1 h. Subsequently, the samples were probed with primary antibodies against ELK1 (#51398, 1:1000, Cell Signaling Technology), SLC7A11 (ab307601, 1:1500, Abcam), GPX4 (ab125066, 1:1000, Abcam), ACSL4 (#38493, 1:1000, Cell Signaling Technology), DJ-1 (ab76008, 1:2000, Abcam), RCHY1 (ab189907, 1:1000, Abcam), UPF1 (#9435, 1:1500, Cell Signaling Technology), Nrf2 (ab313825, 1:1000, Abcam), HO-1 (ab189491, 1:2000, Abcam), TFR1 (#13113, 1:1000, Cell Signaling Technology), FTH1 (#4393, 1:1000, Cell Signaling Technology), Lamin B1 (#17416, 1:2000, Cell Signaling Technology), and GAPDH (AC027, 1:1000, ABclonal, Wuhan, China) at 4 °C overnight. The membranes were then incubated with the secondary antibodies at room temperature for 1 h. Finally, the blots were visualized using the ECL kit (Share-Bio, Shanghai, China) and captured by Image Lab software.

### Statistical analysis

Data are presented as mean ± standard deviation (SD) and analyzed by one-way analysis of variance (ANOVA) followed by Tukey’s post hoc test for multiple groups or Student’s *t* test for two groups using GraphPad Prism 5.0 software. The correlation between ELK1 and LINC00839 expression in NPC tissues was evaluated by Pearson correlation analysis. The survival curve was drawn using the Kaplan-Meier method. *P* < 0.05 was considered as statistically significant.

## Supplementary information

Supplementary information

Supplementary materials

## Data Availability

The data used to support the findings of this study are included within the article and supplementary file.
