# Supplementary material for: LINC00839 transcriptionally activated by ELK1 represses ferroptosis in nasopharyngeal carcinoma by regulating UPF1/RCHY1/DJ-1 axis
Source: NPJ Precis Oncol. 2026 May 14;10:293. doi: 10.1038/s41698-026-01467-1 (PMC13408967; doi:10.1038/s41698-026-01467-1)
Supplement: Supplementary file 1 — Supplementary information [file 41698_2026_1467_MOESM1_ESM.pdf]

Supplementary information

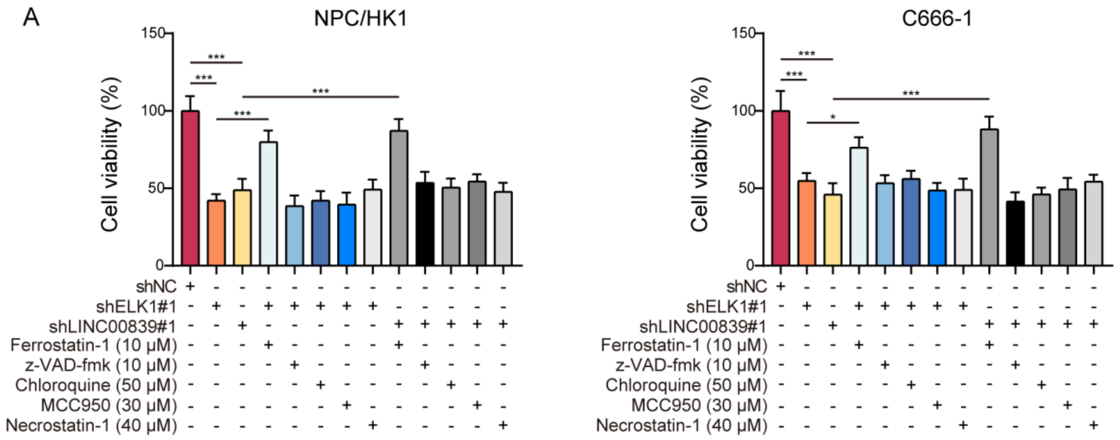

Figure S1. The potential cell death pathways regulated by ELK1 or LINC00839 knockdown in NPC cells.

NPC cells silenced ELK1 or LINC00839 were treated with Ferrostatin-1 (ferroptosis inhibitor, 10 μM), z-VAD-fmk (apoptosis inhibitor, 10 μM), Chloroquine (autophagy inhibitor, 50 μM), MCC950 (pyroptosis inhibitor, 30 μM), or Necrostatin-1 (necroptosis inhibitor, 40 μM) for 24 h. (A) Cell viability was evaluated by CCK-8 assay. *P<0.05 and ***P<0.001.

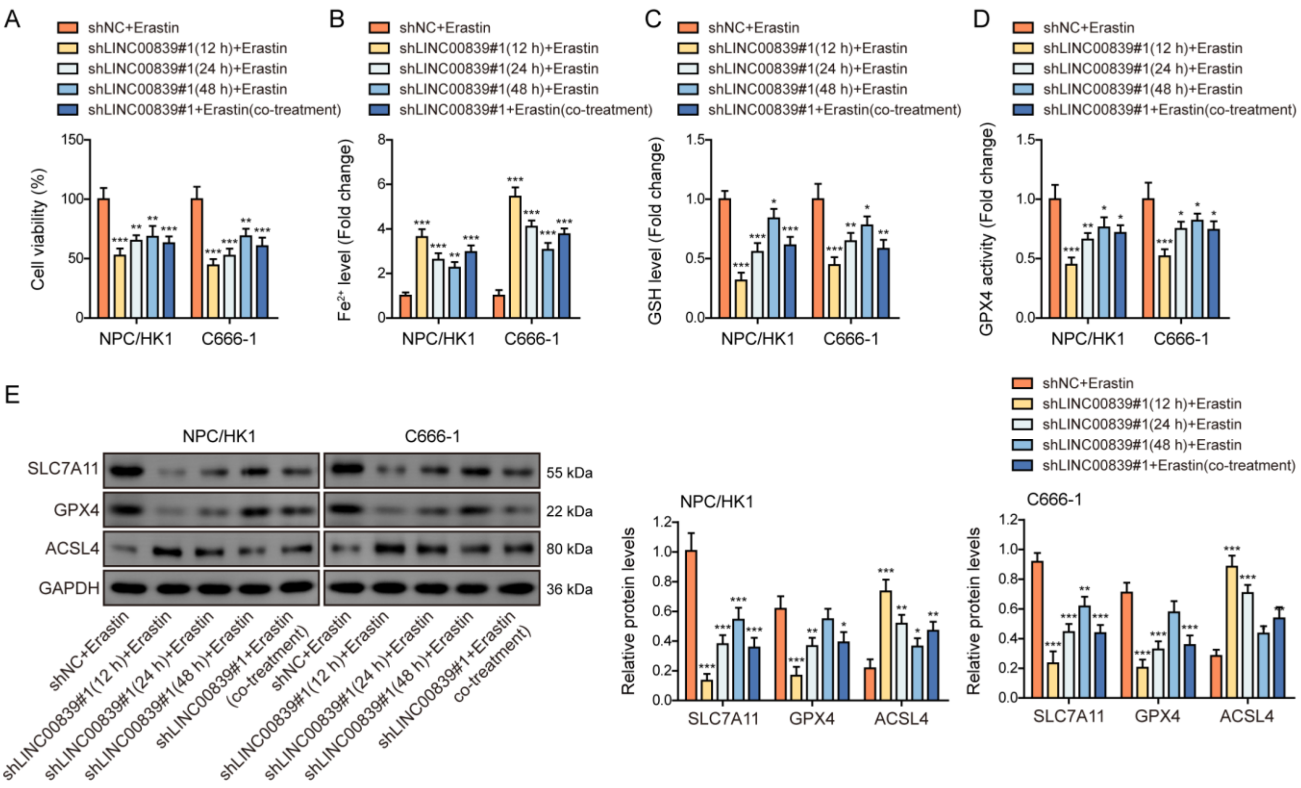

Figure S2 The effects of time-course experiments about LINC00839 knockdown and Erastin treatment on ferroptosis.

NPC cells were pre-transfected with shLINC00839#1 for 12, 24, 48 h followed by treatment with Erastin (10 μM) for 24 h or simultaneous shLINC00839#1 transfection and Erastin (10 μM) treatment for 24 h. (A) Cell viability was analyzed by CCK-8 assay. (B) Fe^{2+} and (C) GSH levels, and (D) GPX4 activity were measured by commercial kits. (E) Protein levels of GPX4, SLC7A11 and ACSL4 were detected by western blotting. *P<0.05, **P<0.01, and ***P<0.001.

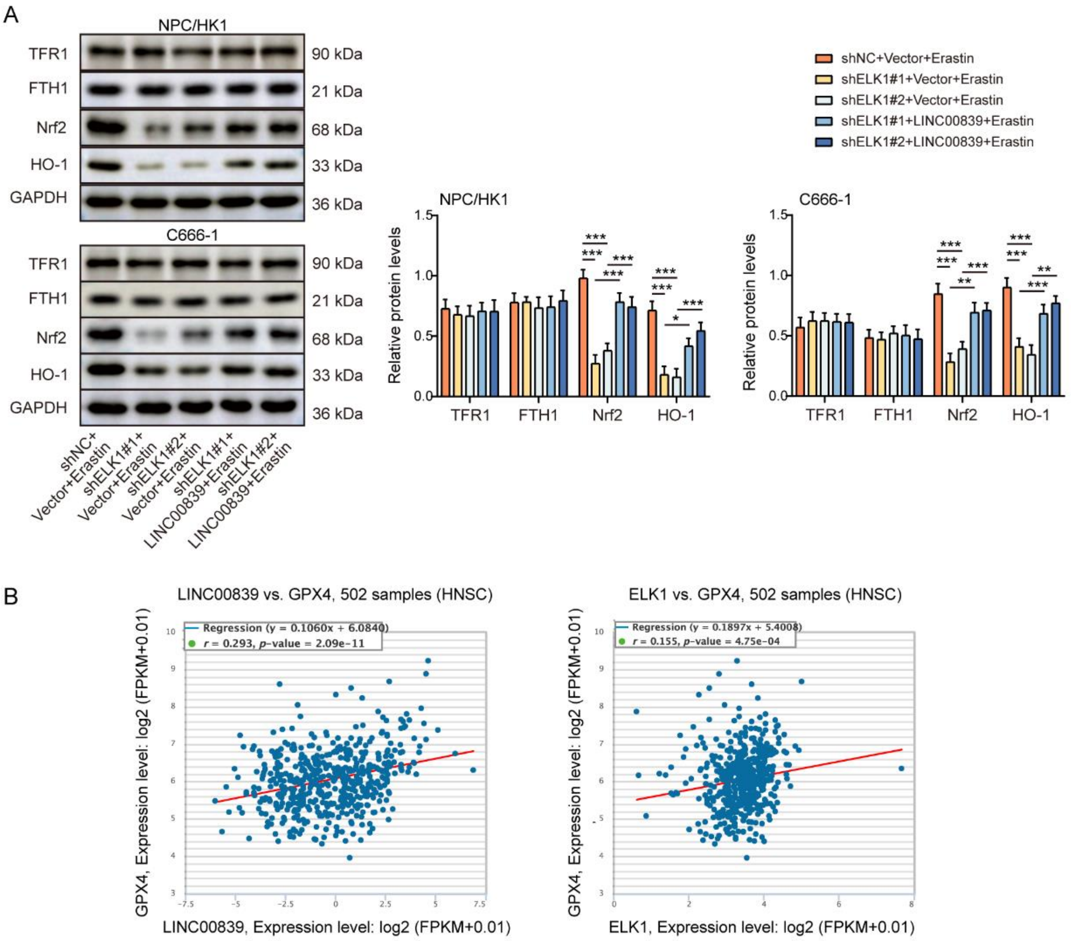

Figure S3. The influence of ELK1/LINC00839 axis on other ferroptosis-related pathways in NPC cells.

NPC cells were transfected with shELK1#1/2 in combination with or without LINC00839 overexpression vector upon Erastin (10 μM) exposure. (A) The protein levels of iron metabolism pathway markers (TFR1 and FTH1), and antioxidant signaling pathway markers (Nrf2 and HO-1) were analyzed by western blotting. (B) The correlation between ELK1/LINC00839 and ferroptosis marker GPX4 in HNSC tissues was analyzed by ENCORI database (n=502). **P*<0.05, ***P*<0.01, and ****P*<0.001.

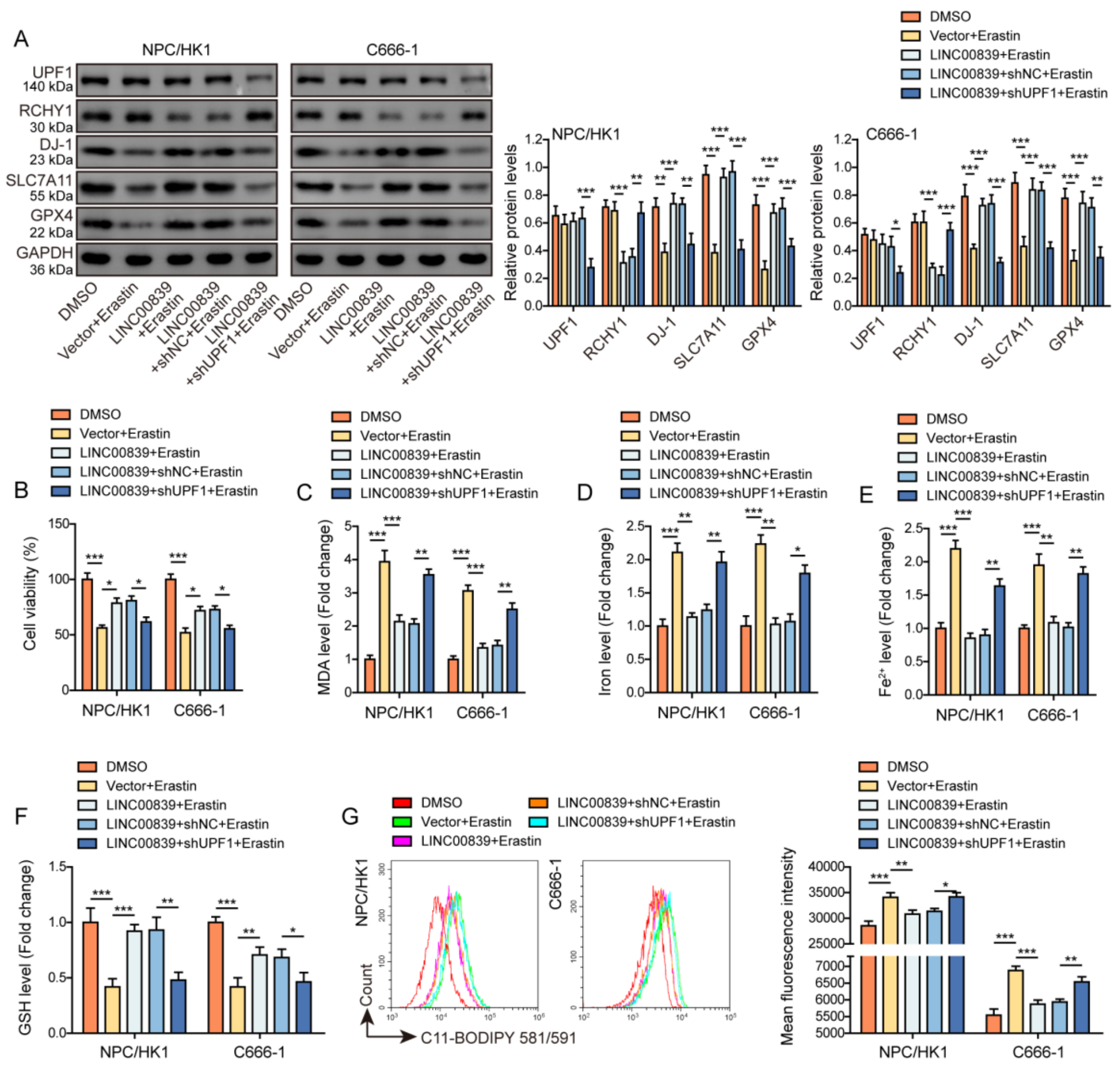

Figure S4. UPF1 was involved in LINC00839-induced ferroptosis resistance of NPC cells.

NPC cells were transfected with LINC00839 overexpression vector together with shUPF1 upon Erastin (10 μM) exposure. (A) Protein levels of UPF1, RCHY1, DJ-1, GPX4, and SLC7A11 were analyzed by western blotting. (B) Cell viability was measured by CCK-8 assay. (C) MDA, (D) iron, (E) Fe^{2+}, and (F) GSH levels, and (G) GPX4 activity were detected by commercial kits. (H) C11-BODIPY 581/591 probe and flow cytometry evaluated the lipid ROS level. **P*<0.05, ***P*<0.01, and ****P*<0.001.

Table S1. Primers sequences of qRT-PCR assay

ELK1	Forward: 5'-CAGCCAGAGGTGTCTGTTACC-3'
	Reverse: 5'-GAGCGCATGTACTCGTTCC-3'
LINC00839	Forward: 5'-GTGGGCTTTCCGCATTTCAC-3'
	Reverse: 5'-TGCATGAGGCAGCTCCAAAG-3'
LINC00665	Forward: 5'-AGGTGCAAAGTGGGAAGTGT-3'
	Reverse: 5'-AGGAAACTACATCAGCGCCA-3'
C5orf66-AS1	Forward: 5'-ATGTAGCGAACCAGCAGGGG-3'
	Reverse: 5'-CTGGCTCAGGTTTCGGCTC-3'
LINC01094	Forward: 5'-CATGGCCAGGGGATCTTCAG-3'
	Reverse: 5'-CACGTTTTCCTTTGGGAGGC-3'
LINC00926	Forward: 5'-AGCATCCACGTTGTATCCCC-3'
	Reverse: 5'-CCTGTGTTCAAATGGGCCTG-3'
UPF1	Forward: 5'-ACCGACTTTACTCTTCCTAGCC-3'
	Reverse: 5'-AGGTCCTTCGTGTAATAGGTGTC-3'
RCHY1	Forward: 5'-TGTGGAATTTGTAGGATTGGTCC-3'
	Reverse: 5'-CAACACGGGATGTGTGAATGT-3'
DJ-1	Forward: 5'-GTAGCCGTGATGTGGTCATTT-3'
	Reverse: 5'-CTGTGCGCCCAGATTACCT-3'
GAPDH	Forward: 5'-CCAGGTGGTCTCCTCTGA-3'
	Reverse: 5'-GCTGTAGCCAAATCGTTGT-3'
