# Supplementary material for: LINC00839 transcriptionally activated by ELK1 represses ferroptosis in nasopharyngeal carcinoma by regulating UPF1/RCHY1/DJ-1 axis
Source: NPJ Precis Oncol. 2026 May 14;10:293. doi: 10.1038/s41698-026-01467-1 (PMC13408967; doi:10.1038/s41698-026-01467-1)
Supplement: Supplementary file 2 — Supplementary materials [file 41698_2026_1467_MOESM2_ESM.pdf]

Raw data of western blot

Figure 1

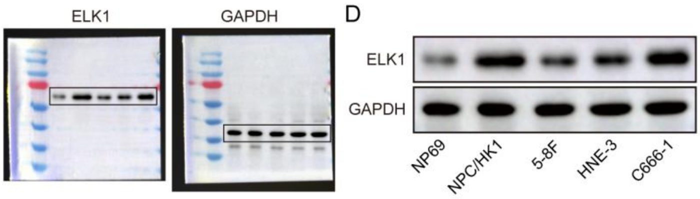

Figure 2

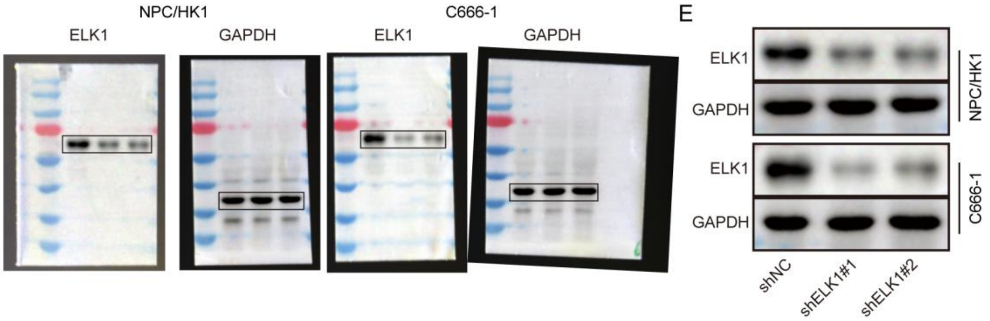

Figure 3

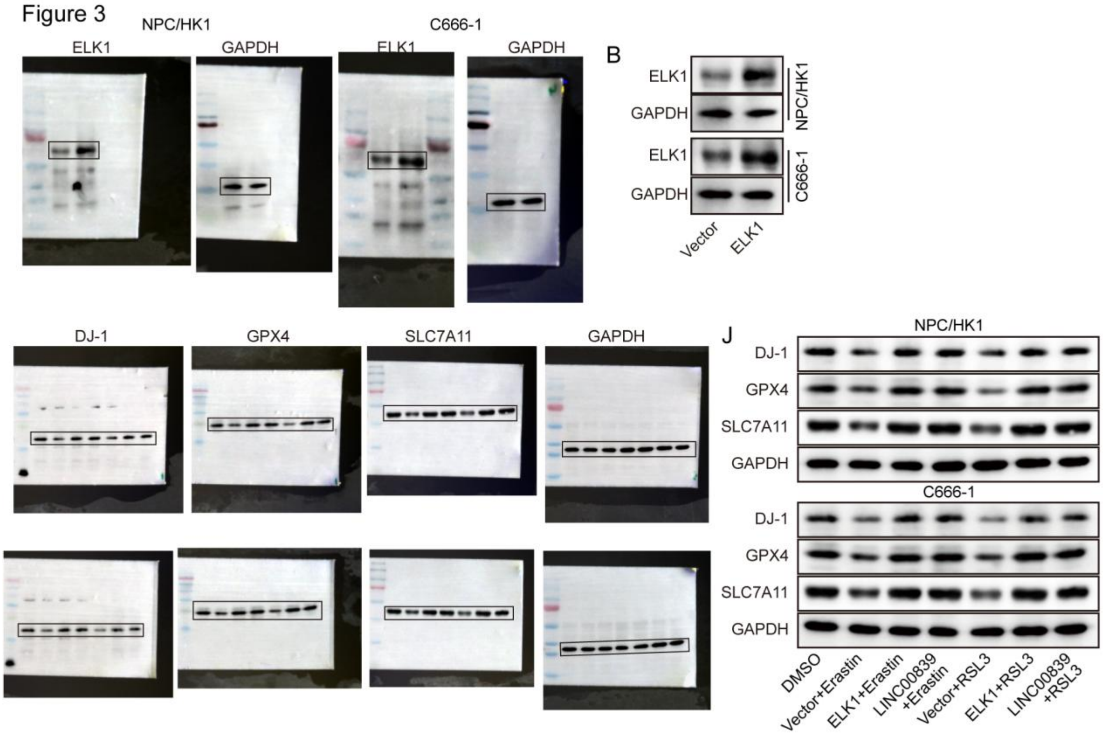

Figure 4

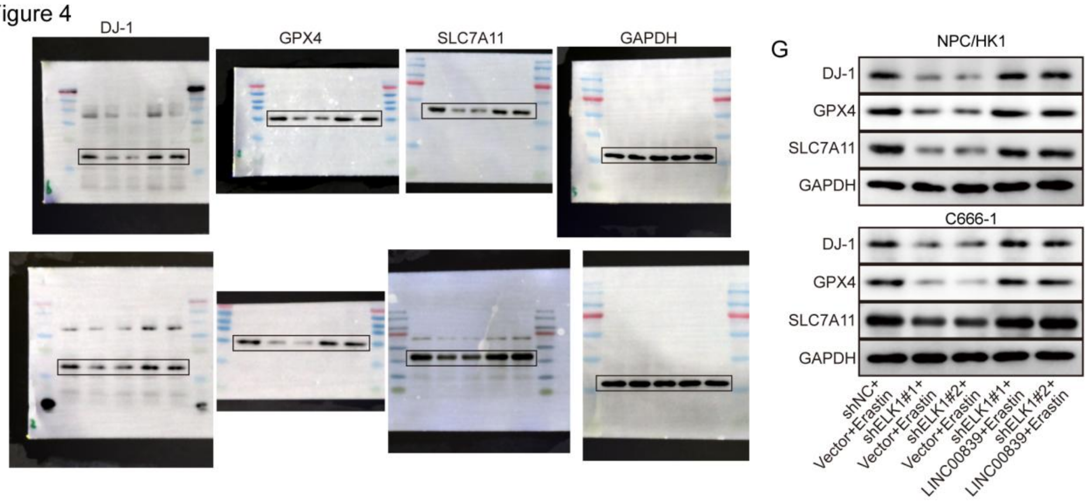

Figure 5

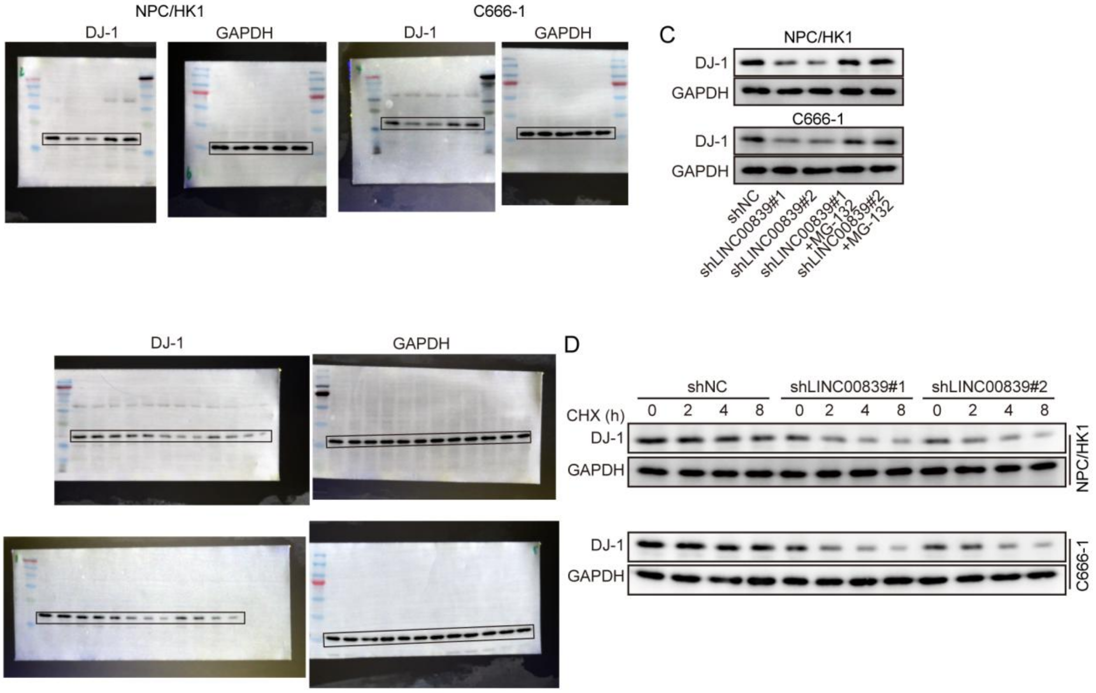

Figure 5

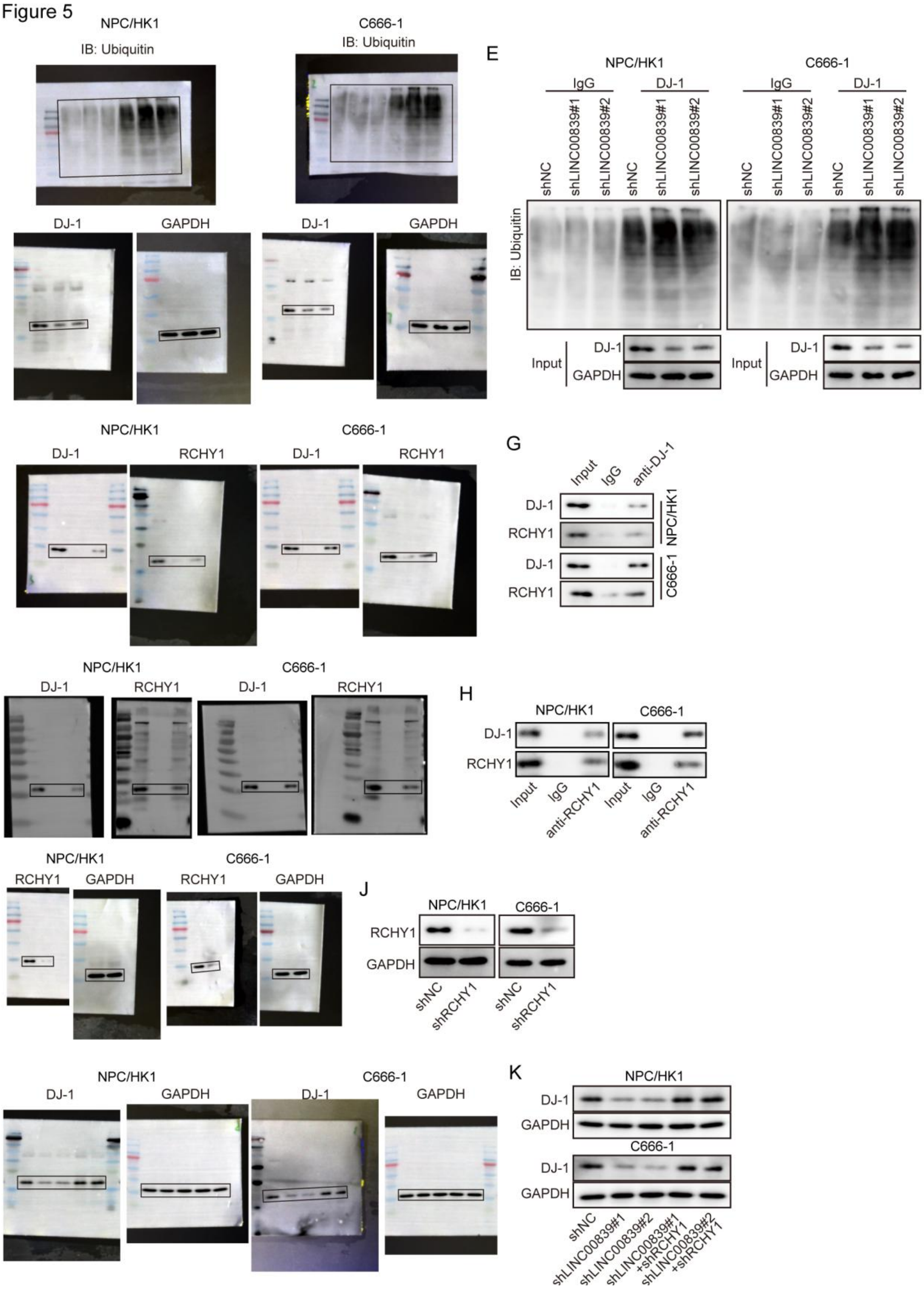
NPC/HK1
IB: Ubiquitin
C666-1
IB: Ubiquitin
DJ-1
GAPDH
E
IgG
shNC
shLINC00839#1
shLINC00839#2
IB: Ubiquitin
Input
NPC/HK1
RCHY1
G
anti-DJ-1
H
anti-RCHY1
J
shRCHY1
K
shLINC00839#1 +shRCHY1
shLINC00839#2 +shRCHY1

Figure 6

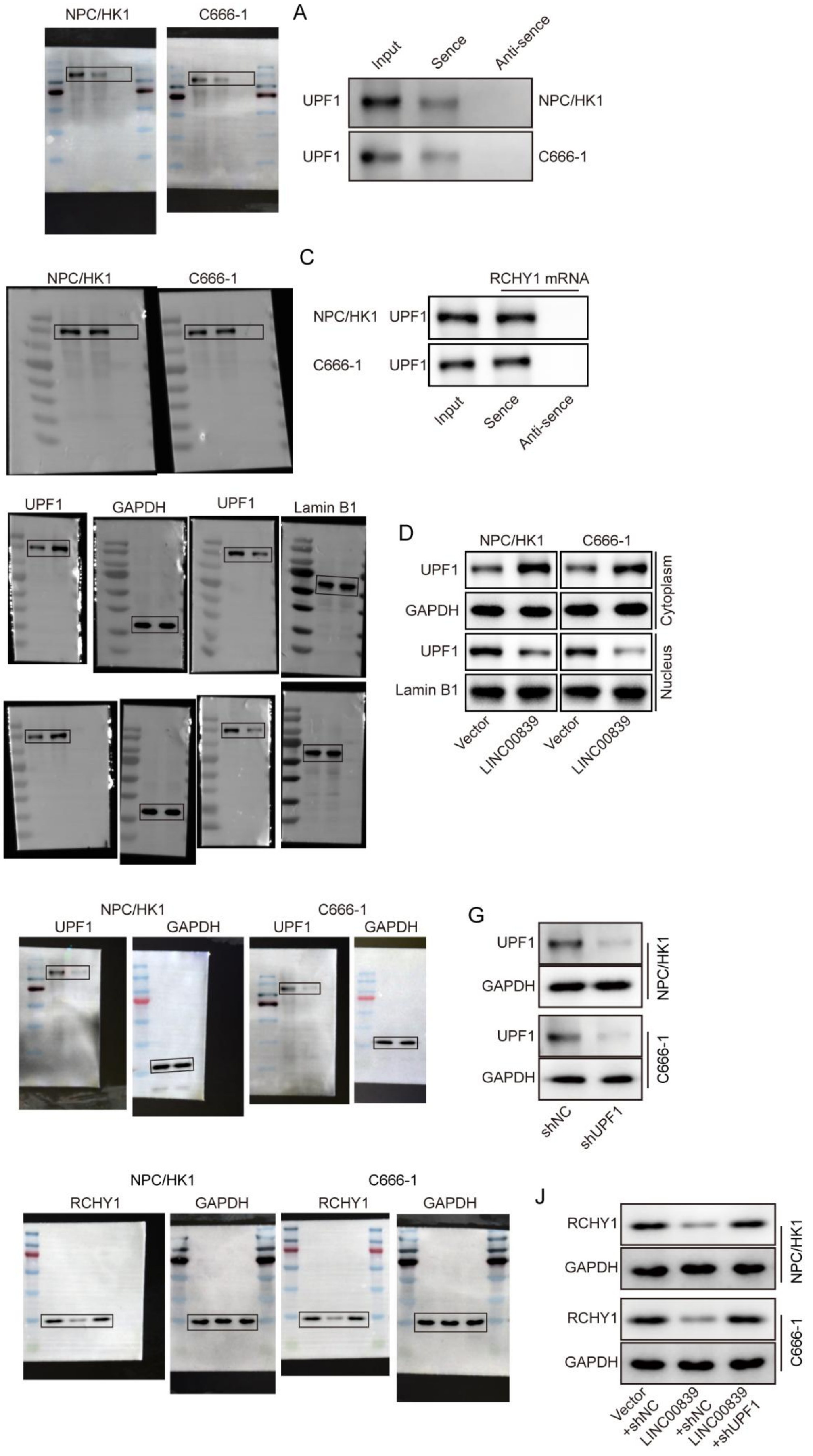
A
NPC/HK1
C666-1
Input
Sence
Anti-sence
UPF1
NPC/HK1
UPF1
C666-1
C
NPC/HK1
C666-1
RCHY1 mRNA
NPC/HK1 UPF1
C666-1 UPF1
Input
Sence
Anti-sence
UPF1
GAPDH
UPF1
Lamin B1
D
NPC/HK1
C666-1
UPF1
GAPDH
Cytoplasm
UPF1
Lamin B1
Nucleus
Vector
LINC00839
Vector
LINC00839
NPC/HK1
C666-1
UPF1
GAPDH
UPF1
GAPDH
G
UPF1
GAPDH
NPC/HK1
UPF1
GAPDH
C666-1
shNC
shUPF1
NPC/HK1
C666-1
RCHY1
GAPDH
RCHY1
GAPDH
J
RCHY1
GAPDH
NPC/HK1
RCHY1
GAPDH
C666-1
Vector +shNC
LINC00839 +shNC
LINC00839 +shUPF1

Figure 7

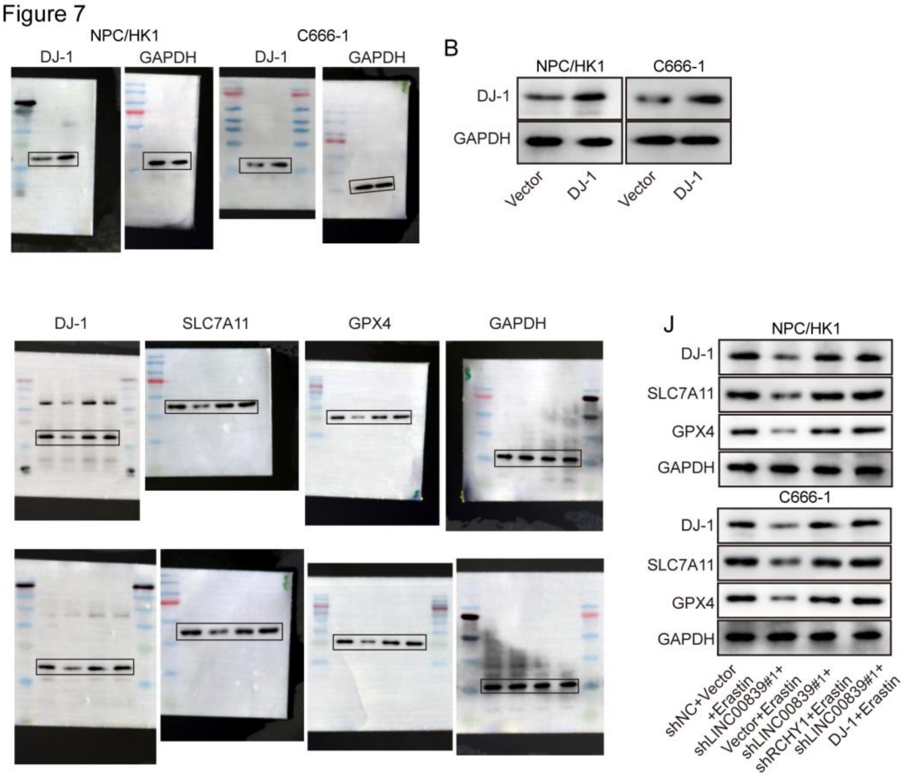

Figure 8

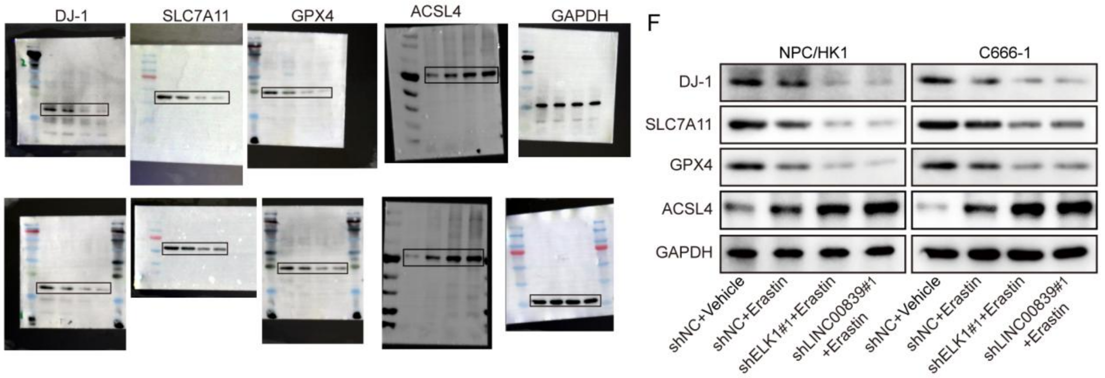

Figure 9

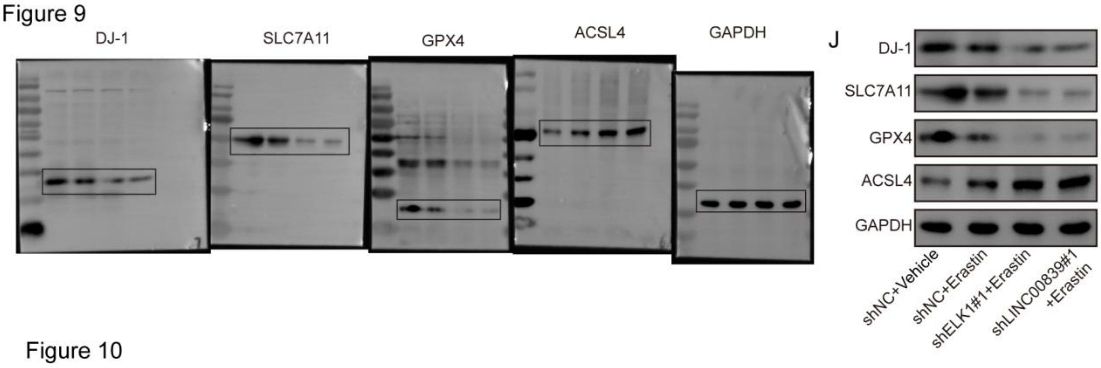

Figure 10

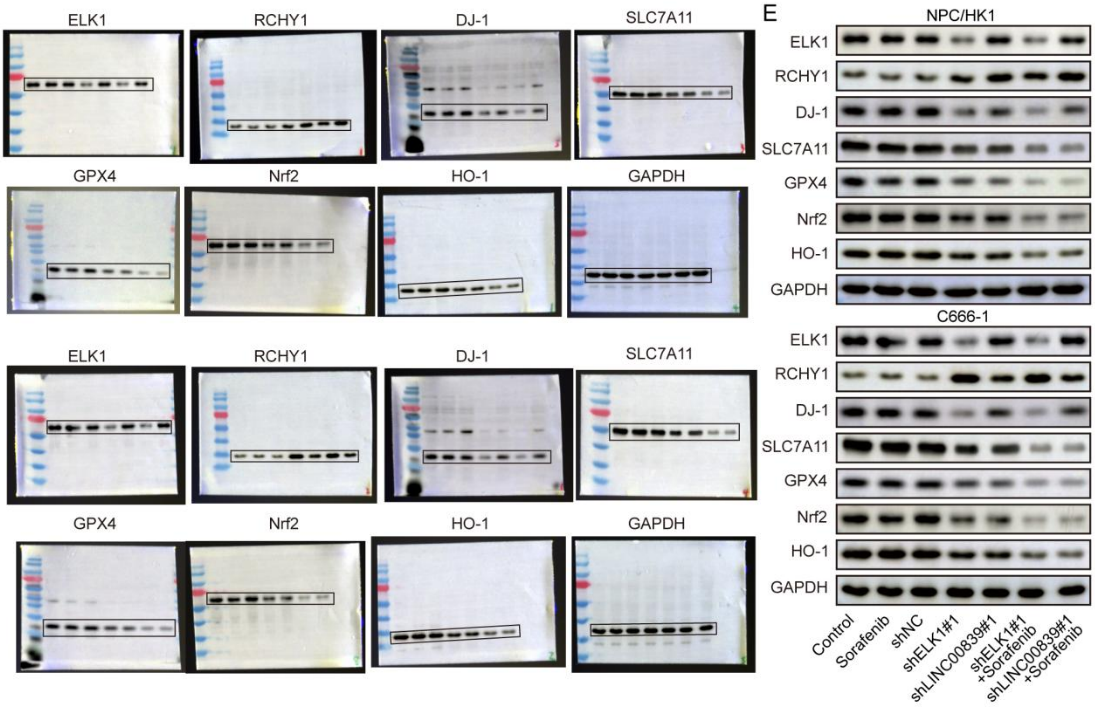

Figure S2

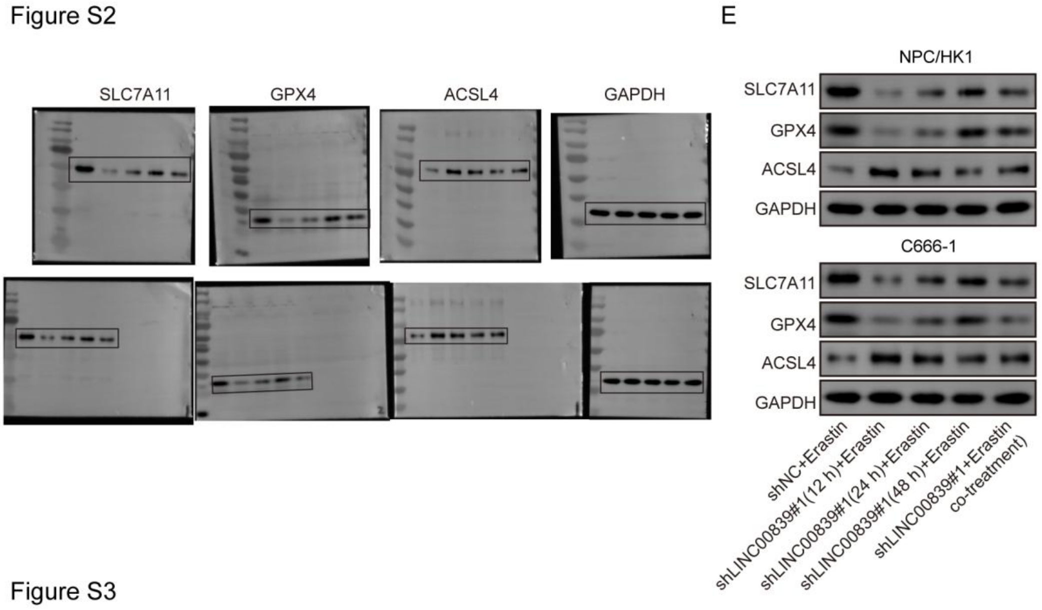

Figure S3

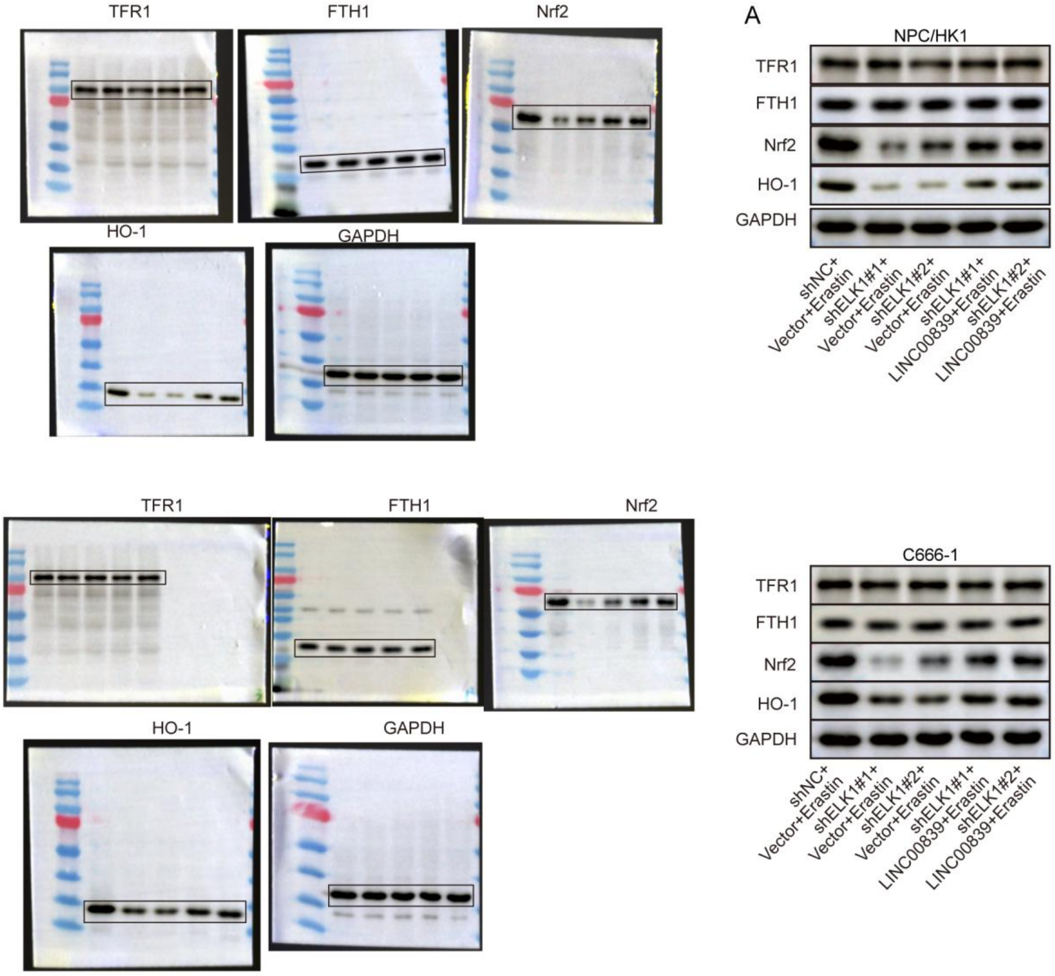

Figure S4

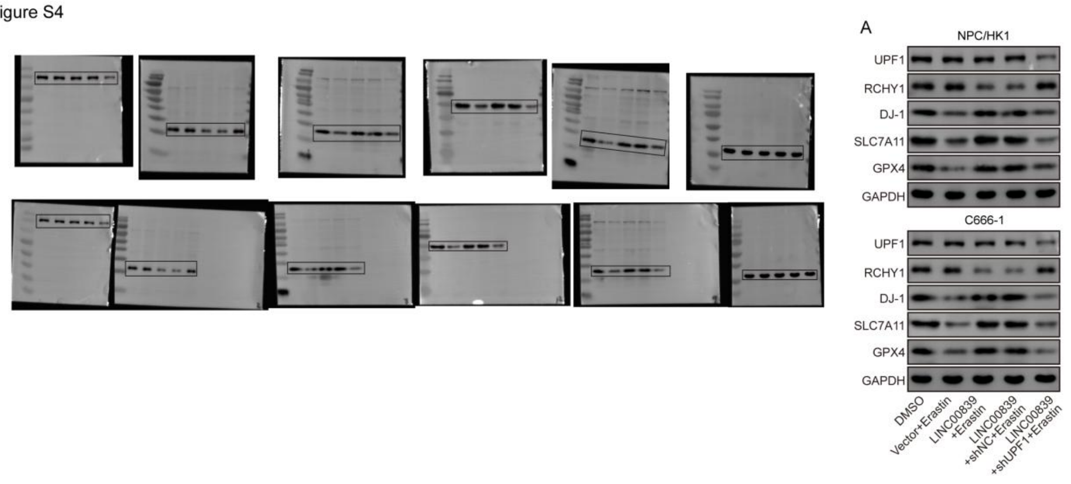

The markers used in this study

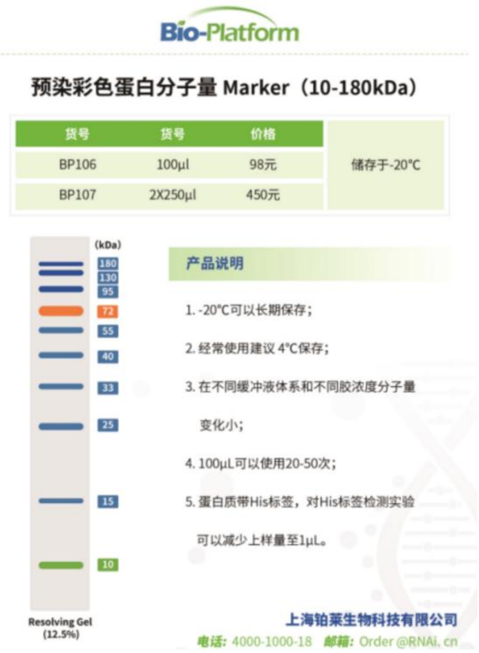

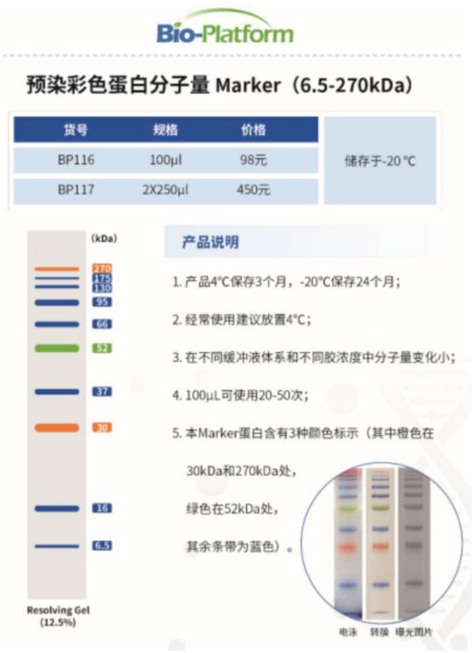

SMOBiO®
Small Bio, Smart Tool

ExcelBand™

Enhanced 3-color Regular Range Protein Marker

PM2510

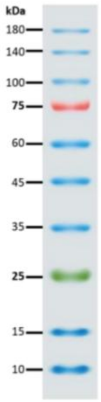

Note. The apparent molecular weight (kDa) of each protein has been determined by calibration against an unstained protein standard; supplemental data should be considered for more accurate adjustments in different electrophoresis conditions.

P06-F11-A1
